# Modeling the integration of bacterial rRNA fragments into the human cancer genome

**DOI:** 10.1186/s12859-016-0982-0

**Published:** 2016-03-21

**Authors:** Karsten B. Sieber, Pawel Gajer, Julie C. Dunning Hotopp

**Affiliations:** Institute for Genome Science, University of Maryland School of Medicine, Baltimore, MD 21201 USA; Department of Microbiology and Immunology, University of Maryland School of Medicine, Baltimore, MD 21201 USA; Greenebaum Cancer Center, University of Maryland School of Medicine, Baltimore, MD 21201 USA

**Keywords:** DNA integration, Somatic genome, Genome variation, Cancer genomics, Host-bacteria interactions

## Abstract

**Background:**

Cancer is a disease driven by the accumulation of genomic alterations, including the integration of exogenous DNA into the human somatic genome. We previously identified *in silico* evidence of DNA fragments from a *Pseudomonas-like* bacteria integrating into the 5′-UTR of four proto-oncogenes in stomach cancer sequencing data. The functional and biological consequences of these bacterial DNA integrations remain unknown.

**Results:**

Modeling of these integrations suggests that the previously identified sequences cover most of the sequence flanking the junction between the bacterial and human DNA. Further examination of these reads reveals that these integrations are rich in guanine nucleotides and the integrated bacterial DNA may have complex transcript secondary structures.

**Conclusions:**

The models presented here lay the foundation for future experiments to test if bacterial DNA integrations alter the transcription of the human genes.

**Electronic supplementary material:**

The online version of this article (doi:10.1186/s12859-016-0982-0) contains supplementary material, which is available to authorized users.

## Background

The advent of next generation sequencing has enabled the interrogation of the human genome and transcriptome with base pair resolution. This new window into the human genome has expanded our understanding of important somatic genome variants such as single nucleotide polymorphisms [1–4], chromosomal rearrangements [5–7], and exogenous DNA integrations [8, 9]. These variants have primarily been identified using paired-end sequencing, a process that relies on breaking the genomic DNA into many small fragments and sequencing each fragment of DNA from the ends inward toward the center. The resulting pairs of sequencing reads share a unique relationship as they originated from the same randomly sheared DNA fragment.

**Fig. 1:**
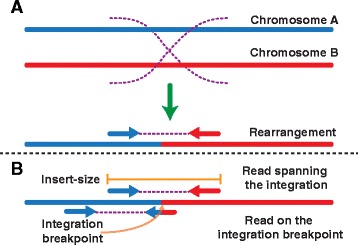
A brief schematic illustrates using next generation sequencing to identify structural variation. A recombination event is illustrated between two chromosomes (**a**). In order to identify structural variation, paired-end reads are identified that map to different chromosomes indicating a fusion of the two different chromosomes has occurred (**b**)

By leveraging the unique relationship between the paired-end reads, chromosomal rearrangements and integrations of exogenous DNA can be identified and characterized [6]. For example, a rearrangement between two chromosomes would result in at least one chimeric chromosome (Fig. 1). By mapping the sequencing reads to a reference genome, a subset of the paired-end reads will support the chromosomal rearrangement by spanning the break point between the two chromosomes with one of the paired-end reads mapping to chromosome A, while the respective other paired-end read maps to chromosome B (Fig. 1). In addition to identifying rearrangements, this technique has also been applied to identify integrations into the human genome of: transposable elements [10–12], viral genomes [8, 9], and bacterial DNA [13]. If the integration site can be rebuilt *in silico*, the structure and sequence of the integration site can be used to determine a potential mechanism of integration [14, 15], elucidate potential functional implications of novel integrations, and lay the foundation for future experiments.

Previously, we identified paired-end reads supporting the integration of *Pseudomonas-like* rRNA genes into the 5′-UTR of genes in human stomach adenocarcinoma (STAD) genomes using RNA-Seq data from The Cancer Genome Atlas (TCGA) [13]. These paired-end reads have one read mapping exclusively to the 16S or 23S rRNA genes of a *Pseudomonas-like* bacteria [13], while the paired read maps uniquely to the 5′-UTR of the human *CEACAM5*, *CEACAM6*, *CD74*, or *TMSB10* genes. As such, these read pairs support bacterial DNA integrations by spanning the junction of the bacterial and human DNA. To identify these regions in the human genome, >4× human coverage in a single sequencing run was required. Despite this level of coverage, single reads that traverse the integration site were not identified, likely owing to the length of the sequencing reads and limits of alignment algorithms. Such reads would enable the assembly of the integration with the base pair resolution needed to determine the potential mechanism of integration [5]. Instead, here, we have used the paired-end reads to model the most likely structure of the integration of *Pseudomonas-*like rRNA gene fragments into *CEACAM5*, *CEACAM6*, *CD74*, and *TMSB10.*

## Results

### Establishing the boundaries of the bacterial rRNA integrations

In all cases, reads were only recovered spanning one side of the junction between bacterial and human DNA, likely because the integration is close to the transcriptional start site. Despite numerous attempts, the integrations could not be assembled using the original reads supporting an integration event or with additional reads located near the integrations that were identified using either alignment- or BLAST-based algorithms. Neither split reads, those that are part bacterial and part human on the breakpoint of the integrations, nor soft-clipped reads could be identified in the original alignments or in alignments to a custom reference with both the human genome and the *Pseudomonas* 16S & 23S rRNA genes. Further BLAST-based examination of the unmapped read in read pairs that had only one read aligned to the human genome in the region flanking the integration or the *Pseudomonas* rRNA gene reference near the integration also failed to identify split reads.

To examine this further, a bacterial-human DNA integration was constructed with the bacterial DNA directly abutting the human DNA. A mock dataset was created of all 101 possible combinations of 100-bp paired end reads spanning the integration breakpoint in this artificial sequence mimicking a bacterial-human DNA integration. The first read generated was entirely bacterial and ended at the integration breakpoint. Each of the 100 subsequent reads in the mock dataset shifted by 1-bp, such that the dataset included a mock read for every position across the integration beginning with an entirely bacterial read and ending with an entirely human read. The second read in the pair was held constant and corresponded to a sequence 225-bp downstream of the break point. LGTSeek identified only 3 (3 %) reads that cover the breakpoint, none of which were soft-clipped as the differences with respect to mapping were similar to those arising from sequencing errors. Therefore, we conclude that LGTSeek, and more specifically the version of BWA used in LGTSeek, is unable to identify reads that span the junction between bacterial and human DNA in this data set.

Given that the bacterial DNA integrations could not be assembled, the focus shifted to estimating the location of the bacterial rRNA gene fragment integrations into the human genome by examining the structure of the human transcript and the reads supporting the bacterial DNA integrations. The integration breakpoint must be downstream of the transcriptional start site (TSS) of each human gene for three reasons. First, the integrations were identified in an RNA-Seq data set derived from transcripts so they must be within the transcript boundaries. Second, examination of the expression of these genes across all participants from the STAD and Breast Cancer (BRCA) data sets from TCGA data available for download from the SRA between September 18^th^–20^th^, 2011 [13] are consistent with an accurately annotated TSS (Additional file 1: Figure S1). Third, the introns and other noncoding regions of the human genome do not have low-level sequencing coverage that would suggest the presence of contaminating genomic DNA. Therefore, the integration must be downstream of the annotated TSS and the TSS is the left-most boundary for the possible location of bacterial DNA integration, relative to the direction of transcription.

The right-most boundary for the bacterial DNA integration break point can be delineated from the position of human reads supporting the bacterial DNA integrations. More specifically, the bacterial DNA integration must be upstream of the human reads supporting the bacterial DNA integration, relative to the direction of transcription. Therefore, the left most boundary for the site of integration in the human genes, in all cases, should be considered the TSS, while the right most boundary position for the integration is the left most position of the consensus sequence for the human reads supporting the bacterial DNA integration.

### Using the library insert-size to refine the location of bacterial DNA integration

The library insert-size and its distribution can be used to refine the location of the bacterial rRNA gene fragment integration. In order to model the integrations with the greatest resolution and accuracy, two calculations were used to determine the number of bases needed between the bacterial and human consensus sequences to mirror the library insert-size distribution.

**Fig. 2:**
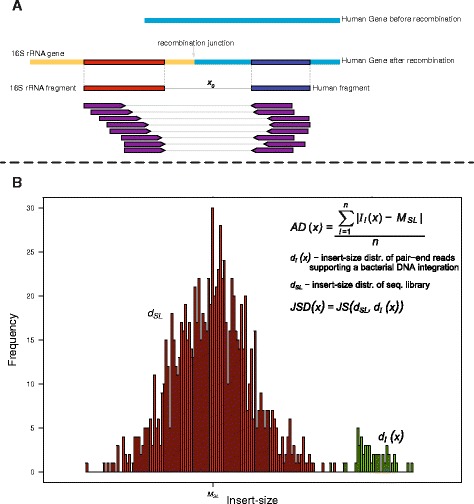
The location of the bacterial DNA integration was refined using the library insert-size. In this hypothetical case, the paired-end reads support the integration of a fragment of the bacterial 16S rRNA gene into a human gene (**a**). Using the consensus sequence of the two fragments, the number of bases, *xx*, needed between the two fragments was titrated so that the insert-sizes of the reads supporting the integration most closely resemble those of the sequencing library. Two calculations were used to determine the optimum distance between the two fragments. The first calculation identified the number of bases (*x*) yielding the minimum average difference (AD) between insert-size of the reads supporting the integration, *I*_***i***_(*x*), and the median sequencing library insert-size, *M*_***SL***_. The second calculation determined the number of bases (***x***) resulting in the minimum Jensen-Shannon distance (JSD) for the insert-size distributions of the sequencing library, *d*_***SL***_, and the reads supporting the integration, *d*_***I***_(*x*) (**b**)

Suppose that the distance between the bacterial and human fragments is *x*, where 0 ≤ *x* ≤ 100 (Fig. 2a), and that each integration has *n* total reads supporting it. The insert-size, *I*_*i*_ (*x*), of the *i*^*th*^ read pair spanning the junction is calculated based on the known positions of each read within the bacterial and human fragment consensus sequences and the assumed distance, *x*, between these fragments. The mean absolute value, *AD*(*x*), of the differences between the median sequencing library insert-size, *M*_*SL*_, and the read pairs supporting the integration can be calculated as $AD(x) = \frac{{\displaystyle {\sum}_{i=1}^n}\left|{I}_i(x)-{M}_{SL}\right|}{n}$, where *n* is the total number of reads supporting the integration. The location of the junction between bacterial rRNA and the human gene of interest was estimated to be within the range, [*L* − *x*_*AD*_, *L*], where *L* is the position of the left end of the human fragment consensus sequence within the human gene and *x*_*AD*_ is the value of *x* which has the minimum value of *AD*(*x*) (Fig. 2b).

The second approach of estimating the distance between the bacterial and human consensus sequences uses a dissimilarity measure called the Jensen-Shannon distance, *JSD*(*x*), between the insert-size distributions of the sequencing library, *d*_*SL*_, and reads supporting the integration, *d*_*I*_(*x*) (Fig. 2b). The Jensen-Shannon distance between two distributions takes values between 0 and 1, with 0 implying the identity of the distributions and 1 indicating major discordance between the distributions. As in the average distance case, the location of the junction between bacterial 16S rRNA fragment and the human gene of interest was estimated to lie within the range, [*L* − *x*_*JS*_, *L*], where *L* is the position of the left end of the human fragment consensus sequence within the human gene and *x*_*JS*_ is the distance value for the minimum value of *JSD*(*x*) (see methods for further details). The majority of the time the AD and JSD calculations were in agreement. When the two calculations differed, the JSD was preferred since it accounts for the distribution of insert size values for the two populations.

### Validating the method

**Fig. 3:**
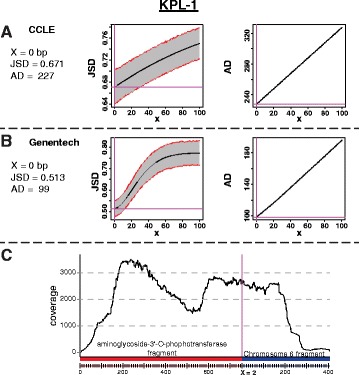
A bacterial DNA integration into the KPL-1 cell line genome was modeled using the JSD and AD calculations. Based on the data from the Cancer Cell Line Encyclopedia [16] and a similar data set from Genentech [17], the JSD and AD methods predicted that the bacterial & human consensus sequences are 0 bp apart (**a**, **b**). However, PCR amplification and sequencing revealed an additional 2 bp of sequence between the two consensus sequences as illustrated (**c**). Consistent with this, a plot of the sequence coverage of the reads across the junction is relatively constant (**c**). No further insertions or deletions are observed in the underlying reads (data not shown)

To validate this method of modeling the bacterial DNA integrations, a bacterial DNA integration in a cancer cell line genome was identified, modeled, and experimentally validated. Using the Cancer Cell Line Encyclopedia [16] and a similar cancer cell line sequencing project that is publicly available through Genentech Inc [17], paired-end RNA-Seq reads were identified that support the likely in vitro integration of a kanamycin resistance gene (aminoglycoside-3'-O-phosphotransferase) near the 67,142 kbp position in chromosome 6 of the KPL-1 cell line. The paired-end reads supporting this integration spanned the junction of bacterial and human DNA at the 3' - side of the kanamycin gene, with respect to the direction of transcription. Given that this integration was likely in vitro, a viral promoter is expected to drive the kanamycin gene expression, which would prevent the bacterial sequence focused pipeline from detecting the 5' - junction between the integrated bacterial DNA and human chromosome. In both independently sequenced datasets, the JSD and the AD predicted that there should been 0 bp between the consensus bacterial and human DNA (Fig. 3a, b). Subsequently, the genomic DNA of the KPL-1 cell line was acquired from Leibniz Institute DSMZ and the junction between the integrated kanamycin resistance gene and chromosome 6 was PCR amplified, cloned, and sequenced. The sequencing revealed that the JSD method correctly predicted the sequence and approximate position of the integration site, but that the exact position was off by 2 bp, with the actual position of integration being 2 bp from that predicted by the model (Fig. 3c).

### KPL1 breakpoint

While investigating the KPL1 integration, soft-clipped reads at the KPL1 bacteria-human DNA junction were identified manually. To better understand why these reads were not identified by the LGTSeek pipeline, a mock dataset was created of all 101 possible combinations of 100-bp paired end reads spanning the cloned and sequenced integration breakpoint. The first read generated was entirely bacterial and ended at the integration breakpoint. Each of the 100 subsequent reads in the mock dataset shifted by 1-bp, such that the dataset included a mock read for every position across the integration beginning with an entirely bacterial read and ending with an entirely human read. The second read in the pair was held constant and corresponded to a sequence 190-bp downstream of the break point, which was selected based on the insert-size distribution of the CCLE data. No split reads were identified from running the entire KPL1 dataset spiked with this mock dataset through the LGTSeek pipeline due to a 24-bp inversion in the bacterial DNA near the KPL1 breakpoint (Additional file 2: Figure S2).

### Bacterial rRNA gene integrations into *CEACAM5 & CEACAM6*

**Fig. 4:**
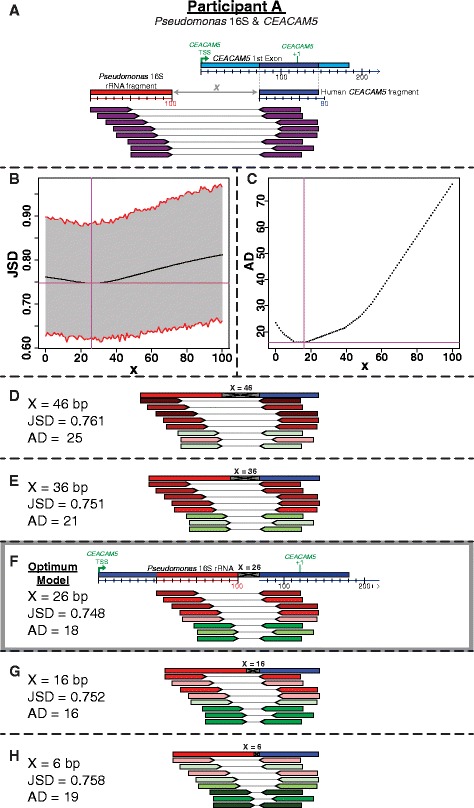
The model of the *Pseudomonas* 16S rRNA gene fragment integration into *CEACAM5* of participant A is presented. The structure of the *Pseudomonas* 16S rRNA gene fragment, the first exon *CEACAM5,* and the paired reads supporting the integration of the rRNA gene fragment into *CEACAM5* are illustrated (**a**). Calculations using the JSD (**b**) and AD (**c**) support that there are 26 bp between the *Pseudomonas* 16S rRNA & *CEACAM5* fragments and that the integration is in 48–73 bp of *CEACAM5* (**f**). The *CEACAM5* sequence upstream of the bacterial 16S rRNA fragment is for illustrative purposes only (**f**). The insert-size for each paired-end read is color-coded with lighter colors being closer to the median insert-size, red designating those insert-sizes larger than the median, and green for insert-sizes less than the median as further clarified in Additional file 7: Figure S7. The optimum model for the structure of the integration (**f**) is compared to alternative distances (x) between the two fragments in (**d**-**h**), where an additional 1.0, 0.5, −0.5, & -1.0 median absolute deviations (20 bp) are placed between the two fragments represented by the gray region. The actual DNA sequence of the gray region is unknown

From the TCGA STAD data set that was previously analyzed [13], two participants have paired-end reads supporting *Pseudomonas* DNA integrating into exon 1 of *CEACAM5* [GenBank:NM_004363.3]. In participant A, the integration of the *Pseudomonas* 16S rRNA gene [GenBank:M34133.1] fragment is supported by 8 paired-end reads (Fig. 4a). Using the JSD, the calculated distance between the bacterial and human fragment consensus sequences is 26 bp, with a minimum JSD value of 0.748 (Fig. 4b), while the AD placed it between 11–17 bp (Fig. 4c). A visual inspection of the read insert-size distribution over a variety of values of *x* (Fig. 4d-h) further supports the JSD prediction. Therefore, given that the human fragment consensus sequence starts at position 74 in the *CEACAM5* gene, the bacterial DNA integration is estimated to occur between positions 48–73 in the *CEACAM5* gene (Fig. 4f).

**Fig. 5:**
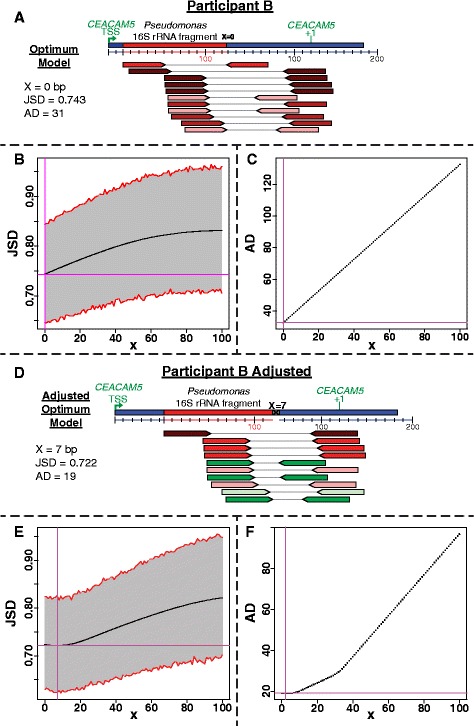
A model of the *Pseudomonas* 16S rRNA gene fragment integration at the 18 bp of *CEACAM5* from participant B is illustrated (**a**). Based on the JSD (**b**) and AD (**c**) calculations, the model has 0 bp between the bacterial and human fragment consensus sequences. After removing the potential outlier paired-end read, the optimum structure for the integration is illustrated in **d**. By removing the potential outlier read, both the JSD and AD decreased (**e** & **f**), supporting that there are 7 bp between the two fragments. This indicates the *Pseudomonas* 16S rRNA likely integrated between positions 45–53 bp of *CEACAM5*

Participant B has 11 paired-end reads supporting the *Pseudomonas* 16S rRNA gene integration into *CEACAM5* (Fig. 5a). Both the JSD and the AD calculations are in agreement that the distance between the two fragments is 0 bp (Fig. 5b, c), and that the 16S rRNA gene fragment has integrated at the 18 bp in *CEACAM5*. However, manually inspecting the insert-size of the reads suggests that the human read farthest upstream is an outlier that does not fit the insert-size distribution of the other reads (Fig. 5a). After removing this potential outlier, a model of the integration was calculated (Fig. 5d). The JSD and AD support that the distance between the consensus sequences is 7 bp (Fig. 5e, f). In addition, both the JSD and AD decreased (Table 1), suggesting that this may be a more accurate representation of the integration, particularly given that decreasing the number of reads in the analysis typically increases the JSD as described below. Therefore, the integration of the 16S rRNA gene fragment is likely between positions 47–53 bp of *CEACAM5*. For the subsequent comparisons in this manuscript, only this latter model is discussed.

**Table 1:** Summary of the bacterial DNA integration models

Name	Min JSD	JSD dist. bp	Integration site	Min. AD	AD dist. bp	Bacterial fragment	Human fragment
A_16S__C5	0.748	26	48–73	16	11–17	354–454	74–147
A_23S1_C5	0.776	22	51–72	4.33	33	1308–1385	73–145
A_23S2_C5	0.785	32	49–80	12.67	29	1626–1699	81–157
B_16S__C5	0.743	0	18	30.82	0	324–449	19–148
B_16Sa_C5	0.722	7	47–53	19.10	0–6	331–449	54–148
B_23S__C5	0.795	35	59–93	12	30–54	1626–1701	94–145
A_16S__C6	0.768	19	59–77	17.60	17	329–454	78–168
C_16S__C6	0.720	23	50–72	17.89	31	321–456	73–140
D_16S1_CD	0.802	2	180–187	27.50	0–20	563–708	188–278
D_16S2_CD	0.802	41	136–147	26.50	7–60	927–1022	184–241
D_23S__CD	0.815	22	167–195	166.75	0	892–1323	196–306
E_16S1_CD	0.809	48	163–205	46	0–65	589–697	206–290
E_23S1_CD	0.791	31	201–225	0.50	44–45	907–978	226–296
E_23S2_CD	0.782	28	170–191	29	0–38	1255–1379	192–269
F_16S_T10	0.812	19	(−5)–13	169.44	0	701–927	14–196

Names indicate the participant with letters A through F, their respective rRNA gene integration (16S, 23S), and the human gene it has integrated into (C5 = *CEACAM5*, C6 = *CEACAM6*, CD = *CD74*, T10 = *TMSB10*)

If the potential outlier paired-end read is excluded, the modeling suggests that the 16S rRNA integrations into the *CEACAM5* gene of participants A & B are nearly identical (Table 1), with the models only differing by a few bp (354–454 & 331–449 bp). In addition, the human reads from participants A & B map to similar positions in *CEACAM5* (74–147 & 54–148 bp, respectively).

In addition to the 16S rRNA gene integration described above, participant A also has 3 paired-end reads supporting an integration of 1626–1699 bp from the *Pseudomonas* 23S rRNA gene [GenBank:Y00432.1] into the *CEACAM5* gene. The JSD and AD calculations support that the integration is between positions 49–80 of *CEACAM5*. In addition, participant B has 2 reads supporting integration of a nearly identical fragment of the 23S rRNA gene (1626–1701 bp) integrating into an almost identical region in *CEACAM5* (positions 59–93). Therefore, the data support two different *Pseudomonas* DNA integrations into approximately the same position in *CEACAM5* for both participants A & B.

Similar to *CEACAM5*, participants A & C have similar *Pseudomonas* 16S rRNA gene fragment integrations into the related *CEACAM6* [GenBank:NM_002483.6]. Both participants have nearly identical fragments of the 16S rRNA gene (329–454 & 321–456 bp) integrated into *CEACAM6* at the same predicted position (59–77 & 50–72 bp) (Table 1). In addition, these *CEACAM6* integrations are similar to the (a) position of the 16S & 23S rRNA integrations and (b) the sequence of the 16S rRNA integrations into *CEACAM5*.

### *CD74* integrations

Both participants D and E have fragments of the *Pseudomonas* 16S and 23S rRNA integrated in the *CD74* gene [GenBank:NM_001025159.2]. Both participants have almost identical fragments of the 16S rRNA gene (563–708 & 589–697 bp) integrated into overlapping positions in the *CD74* gene. For participant D, the calculated model supports that the 16S rRNA fragment integrated between positions 180–187 bp of *CD74*, while the fragment of 16S rRNA integrated into participant E’s *CD74* gene between positions 163–205 bp (Table 1). The data supporting these two integrations are consistent and suggests that the two participants have similar integrations*.* The data also support a second fragment further downstream in the 16S rRNA gene (927–1022 bp) integrating into positions 136–147 bp of *CD74* in participant D.

There are 3 different fragments of the 23S rRNA gene fragments with support for integration into the *CD74* gene of participants D & E. All three integrations have similar locations in the *CD74* gene (~190-290 bp) (Table 1). Of the three integrations, two integrations, one each from participant D & E, are predicted to have integrated into the same region of *CD74* (167–195 & 170–191 bp). The remaining 23S rRNA gene fragment with support for integration into *CD74* is predicted to have integrated into the 201–225 bp of *CD74* of participant E (Table 1).

### *TMSB10* integrations

Unlike the bacterial DNA integrations into *CEACAM5*, *CEACAM6*, and *CD74*, the *TMSB10* gene [GenBank:NM_021103.3] has only one participant with the integration of a *Pseudomonas* 16S rRNA fragment (701–927 bp). The model estimates that the 16S rRNA fragment integrated into the first 13 bp of *TMSB10* (Table 1).

### Calibrating the JSD

**Fig. 6:**
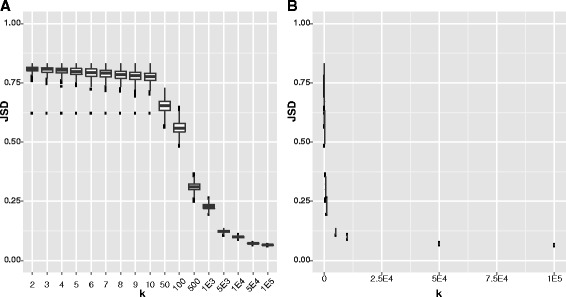
Boxplots illustrate the JSD (*y-axis*) calculated for subsets of RNA-Seq data mapped to the human transcript. The insert-size distribution of the sequencing library of participant B was compared to the distribution of insert-sizes of specific values of randomly selected reads that mapped to the *CEACAM5* transcript from the sequencing library of participant B (**a**). Panel A has a discontinuous x-axis to better illustrate the JSD over the various number of reads tested (k); panel **b** illustrates the same data with a continuous *x-axis*

As described previously, a calculated JSD value of 0 supports that the two populations are identical, while a value of 1 indicates major discordance between the distributions. The average JSD for the models of bacterial DNA integration presented here was 0.78. Therefore, the JSD calculation was investigated further to determine why it was not closer to zero. To test the effect that the number of reads had on the JSD, the JSD was calculated for 1000 samples for specific values of *k*, such that 0 ≤ *k* ≤ 100,000, where *k* represents the number of randomly sampled read pairs that mapped to the *CEACAM5* transcript from the sequencing library of participant B (Fig. 6). Samples with 3–10 randomly selected reads had a JSD of ~0.78, consistent with the results for modeling the integrations. As the number of randomly sampled reads increases, the JSD decreases. The average JSD continues to decrease as the number of reads increases until around 5000 reads when the JSD starts to plateau, eventually reaching a minimum of 0.07 at 100,000 reads, the maximum number tested.

### Guanine rich sequences

While modeling the integrations, it was observed that both the bacterial and human fragment consensus sequences seemed to be particularly rich in guanine. Consistent with this observation, the median %GC for the bacterial and human fragment consensus sequences was 52.7 % and 58.5 %, respectively. The %GC and %AG were compared for the consensus sequences supporting the bacterial DNA integrations using the Wilcoxon rank-sum test (WRT) (Table 2, Additional file 3: Figure S3). The bacterial and human consensus sequences were significantly different than each other (Table 2). Both sets of consensus sequences were also significantly different than the complete RefSeq *Pseudomonas* genomes and the human genomic reference (Table 2). The human fragment consensus sequences were also significantly different that the *Pseudomonas* 16S & 23S rRNA genes available in the Silva database (Table 2). Considering that the predicted integration sites are typically into the first 150 bp of each human transcript, the %GC of the fragment consensus sequences supporting the integrations was compared with the first 150 bp of each transcript from the human transcriptome reference and found that the %GC for the human fragment consensus sequences was significantly different (Table 2). Given that the expressed transcripts may have a different GC profile [18], the %GC for the fragment consensus sequences was compared to the expressed transcripts (>0 RPKM) from the participants with the bacterial DNA integrations. The %GC of the human consensus sequences was significantly different than the expressed transcripts (Table 2). Next, by stratifying the expressed genes into quartiles based on their RPKM, the %GC for the quartiles of expressed transcripts was compared to consensus sequences. The human fragment consensus sequences were significantly different than the two most highly expressed quartiles (Q3 & Q4) (Table 2). In addition, with a less stringent but acceptable significance level of 0.05, the %GC for the lowest two quartiles (Q1 & Q2) are likely significantly different than the human fragment consensus sequences (Table 2).

**Table 2:** Wilcoxon rank-sum test comparison of GC & AG content

References	%GC of human fragment consensus sequences	%GC of bacterial fragment consensus sequences	%AG of human fragment consensus sequences	%AG of bacterial fragment consensus sequences
	*p*-value	*p*-value	*p*-value	*p*-value
Human fragment consensus sequences	NA	9.5e-04*	NA	1.5e-01
Bacterial fragment consensus sequences	9.5e-04*	NA	0.15	NA
*Pseudomonas* 16S rRNA genes	8.3e-06*	7.7e-02	1.0	8.4e-08*
*Pseudomonas* 23S rRNA genes	2.2e-10*	1.0	1.0	5.3e-09*
*Pseudomonas* genomes	6.4e-05*	7.2e-08*	NA	NA
Human genome	6.8e-09*	1.2e-06*	NA	NA
Human transcriptome	8.0e-06*	1.8e-02	1.0	7.3e-02
Human transcriptome first 150 bp	5.7e-05*	5.2e-02	1.0	1.0
Human genes with integrations	8.1e-01	1.0	0.64	7.8e-02
Human genes with integrations first 150 bp	1.0	6.3e-02	1.0	2.6e-01
Expressed human transcripts	2.9e-03*	1.0	1.7e-3*	2.0e-09*
Q1 Expressed human transcripts	1.2e-02	3.5e-01	7.3-e4*	1.4e-09*
Q2 Expressed human transcripts	8.0e-03	6.4e-01	1.1e-3*	1.7e-09*
Q3 Expressed human transcripts	2.1e-03*	6.1e-01	2.5e-3*	2.2e-09*
Q4 Expressed human transcripts	2.7e-04*	2.4e-01	4.4e-4*	3.4e-09*
Expressed human transcripts first 150 bp	1.0	4.3e-01	3.7e-2	4.6e-07*
Q1 Expressed human transcripts first 150 bp	1.0	3.5e-01	1.7e-2	1.3e-07*
Q2 Expressed human transcripts first 150 bp	1.0	6.4e-01	2.8e-2	3.3e-07*
Q3 Expressed human transcripts first 150 bp	1.0	6.1e-01	7.6e-2	1.0e-06*
Q4 Expressed human transcripts first 150 bp	1.0	2.4e-01	4.6e-2	9.7e-07*

* = *p* < 0.005; *NA* not applicable

In addition to GC-content, the enrichment of guanine suggests that these are purine-rich sequences, which have been shown to have a role in regulating transcription [19–21]. Therefore, the percentage of bases that are purines (%AG) for each fragment was calculated using the transcribed strand of each gene and compared to various references using the WRT (Table 2, Additional file 3: Figure S3). The median %AG for the human and bacterial fragment consensus sequences was 55.8 & 61.7, respectively. The %AG of the bacterial fragment consensus sequences was significantly different than the *Pseudomonas* 16S & 23S rRNA gene database, and both of the complete and first 150 bp of the expressed human transcripts in the participants with the bacterial DNA integrations (Table 2, *p* < 0.005, WRT). On the other hand, the %AG of the human fragment consensus sequences were statistically different than the expressed transcripts from the participants with the bacterial DNA integrations (Table 2, *p* < 0.005, WRT).

Due to the observed higher guanine content of these sequences, the human genes with bacterial DNA integrations were investigated for guanine associated motifs. A database search of published G-quartets in the human genome [22] did not reveal evidence for G-quartets near the integrations. Examination of the first exon and 1 kbp upstream of the TSS for *CEACAM5, CEACAM6,* and *CD74* did not reveal CpG islands (Additional file 4: Figure S4A-F). On the other hand, all three algorithms support CpG islands in the 1 kbp region upstream of the *TMSB10* TSS. However, only 2 of the 3 algorithms suggested the CpG island may extend downstream of the TSS near the predicted integration into *TMSB10* (Additional file 4: Figure S4G). CpG islands are typically defined as ≥200 bp regions that have more observed than expected CpG dinucleotides (>0.6) and high GC content (>0.5). Next, the window size for identifying CpG islands was decreased to refine the location of the CpG island signal in the first exon of *TMSB10.* The CpG signal was determined to originate from the 3' end of the first exon of *TMSB10*, while the side of the first exon near the predicted integration site had no CpG signal (Additional file 4: Figure S4H). Therefore, it is unlikely the bacterial integration into *TMSB10* disrupted CpG sites.

### rRNA structure

Based on the position of the bacterial DNA integrations into 5′-UTR of the human genes, it is possible that the bacterial DNA integrations altered the expression of the human genes by disrupting a regulatory element in the 5′-UTR. One reason to expect that this might be the case is that the integrations arise from the 16S & 23S rRNA genes that contain numerous stem-loop structures that have the potential to alter secondary structure and thus transcription. Therefore, the consensus sequence of the integrated bacterial DNA was examined for the presence of stem-loop structures from the bacterial rRNA gene by mapping the bacterial fragments onto the known secondary structure (Additional file 5: Figure S5). It was concluded that 11/14 integrations have large stem-loop structures in the middle of the bacterial fragments; while the remaining three fragments of integrated bacterial DNA have stem-loop structures near the ends of each fragment.

Subsequently, the predicted RNA structure of the human transcripts was examined to determine if the bacterial integrations may have disrupted the secondary structure of the human transcripts. By mapping the predicted position of each integration onto the *CEACAM5*, *CEACAM6*, *CD74*, and *TMSB10* predicted mRNA transcript structures, it was determined that the integrations are either in, or near, complex secondary structures (Additional file 6: Figure S6).

### Relative position of integration

To explore if the bacterial DNA integrations may be occurring in the same relative position within each human gene, the relative position of the predicted site for integration within the first exon was calculated (Table 3). While the *CEACAM5* and *CEACAM6* integrations are enriched around the middle of the first exon, the two genes also share homology and have similar structures. The CEACAM genes, *CD74*, and *TMSB10* all have different relative position of integrations (Table 3).

**Table 3:** Relative position of the bacterial DNA integrations

Name	Absolute distance of the integration from TSS (bp)	Relative distance of the integration from the TSS of 1^st^ Exon (%)
A_16S__C5	61	50
A_23S1_C5	62	51
A_23S2_C5	64	53
B_16S__C5	18	15
B_16Sa_C5	50	41
B_23S__C5	76	62
A_16S__C6	64	45
C_16S__C6	62	41
D_16S1_CD	184	79
D_16S2_CD	160	69
D_23S__CD	182	78
E_16S__CD	185	80
E_23S1_CD	166	72
E_23S2_CD	181	78
F_16S__T10	4	4

## Discussion

### Obstacles in assembling the integrations

Here, the Jensen-Shannon distance between the insert-size distributions of the paired-end reads supporting the *Pseudomonas-like* DNA integrations relative to the respective sequencing library was used to model bacterial DNA integration in *CEACAM5*, *CEACAM6*, *CD74*, & *TMSB10*. The JSD values supporting these models are within the expected range of the JSD values given the number of reads supporting the bacterial DNA integrations.

Ideally, the sequencing reads would be assembled to determine the exact integration site and sequence. There are likely three compounding factors that prevent the assembly of these bacterial DNA integrations. First, the reads are only 51 bp. In order to find a read that is on the integration breakpoint, the read on the breakpoint needs to be able to be split into uniquely human and bacterial portions. As a result of the short read length, a read would have to be exactly half human and half bacterial in order to give a sufficient sequence length of ~25 bp to identify each portion. This is highly unlikely to occur. Second, there is limited coverage of the integrations. The limited coverage is likely due to the proximity of the integrations to the beginnings of transcripts where RNA-Seq coverage is poor. In addition, it is possible that the low RNA-Seq coverage is the result of the bacterial DNA integrations inducing decreased expression. Lastly, the data suggest a heterogeneous population of transcript variants in a tumor that makes *de novo* assembly difficult.

In a mock-experiment with an artificial bacterial-human DNA integration, LGTSeek identified only 3 (3 %) reads that cover the breakpoint. These reads align to the bacterial DNA, with only 1–3 bp at the 3'-end of the read covering the breakpoint. LGTSeek uses a version of BWA that may not identify these reads. While a more recent version of BWA identifies soft-clipped reads better, it also works on the assumption that all reads should match the reference and as such has been found to erroneously align bacterial reads to the human reference. In KPL1, the cloned and sequenced bacteria-human DNA junction was used to generate a similar mock dataset that demonstrates that split and soft-clipped reads could not be identified due to a 24-bp inversion. Given that when the bacterial and human DNA are flush, only 3 % of read pairs are identified, and that there are only 5 read pairs supporting STAD bacterial DNA integrations on average, we do not expect that soft-clipped reads would be identified even if the bacterial DNA is flush to the human DNA. Furthermore, such soft clipped reads that do exist would be difficult to distinguish from sequencing error or sequencing polymorphism between the bacterial sequence and the reference genome. Additionally, the average distance between the bacterial and human fragments of DNA in the models described here was found to be ~24 bp, which suggests that the two may not be flush, further explaining the difficulty in detecting such reads.

### Heterogeneous integrations

Participants A through E all have data supporting multiple integrations of different fragments of the 16S and 23S rRNA *Pseudomonas* gene into the same human gene within each participant. In addition, the data support multiple participants with similar, if not identical, bacterial DNA integrations. For example, participants A & B have 16S and 23S rRNA integrations into the 50–70 bp of *CEACAM5*. This latter result suggests that the tumors sequenced have a heterogeneous population of cancer cells with different integrations.

### Characterizing the integrations

These bacterial DNA integrations were investigated to determine if they are enriched in absolute position as well as relative position within the UTR and first exon of each gene. However, no pattern for the position of integration into these genes was identified, suggesting that each gene may have a different feature that is affected by, or permissive to, the bacterial DNA integrations.

The bacterial and human fragments supporting the integrations are high in guanine, demonstrated by the combination of high %GC and high %AG. Despite the high guanine content, it does not appear that the bacterial DNA integrations disrupted G quartets or CpG islands, but the disruption of other G-rich motifs cannot be ruled out.

### Functional consequences of the bacterial DNA integrations

The integrations of the *Pseudomonas* 16S and 23S rRNA genes may be important and have a biological effect by altering the transcriptional regulation of the human genes. One possibility is that the integration of bacterial DNA introduced new structures at the breakpoint between bacterial and human DNA that alter the transcriptional regulation of these human genes. Given that all of the bacterial integrations have stem-loop structures, secondary structure may play an important role. Alternatively, the integrated bacterial sequence could alter the regulation of these genes by disrupting either the transcript stability, or the availability of the transcriptional and/or translation machinery to bind to these genes.

Considering that the integrations are into the 5'-UTR of these genes, before the translational start site, it is unlikely that the integrations introduced a frameshift or premature stop codon. It is also unlikely that these integrations have interfered with splicing since the integrations are not near a splice site recognition sequence. However, it cannot ruled out that the integrations have created novel transcriptional start sites, translational start sites, or splice sites that would have functional consequences.

## Conclusion

Based on the available data, models were generated for the bacterial 16S and 23S rRNA fragment integrations into the human *CEACAM5*, *CEACAM6*, *CD74*, and *TMSB10*. These mutations are especially intriguing because of their positions in the 5′-UTR near the transcriptional start site. The models presented here lay the groundwork for further in vitro experiments reconstructing these sequences to test if the bacterial rRNA integrations may alter the expression of the human genes.

## Methods

### Library insert-size

The sequencing reads were aligned to the human RefSeq transcriptome reference (available for download 02/22/2013) [23] using BWA v.0.5.9-r16 [24] with the default settings. Picard [25] was used to calculate each participant’s insert-size for the library with default settings. Only the forward-reverse mapped reads were used in the insert-size calculation. The median and absolute deviations of the insert-size were used because the distribution of the data was typically asymmetric. A consistent color scheme (Additional file 7: Figure S7) was used in the figures to illustrate how a given read pairs insert size relates to the distirbution of inserts sizes in the library.

### Average difference

The consensus sequence for the bacterial and human fragments of DNA that support an integration was determined by mapping the appropriate reads to a reference using the farthest upstream and downstream reads to mark the boundaries of each fragment. The consensus sequence of each fragment was determined from the alignment using samtools and bcftools [26, 27]. All the coordinates reported here are based on the alignment of the reads to: *Pseudomonas* 16S rRNA [GenBank:M34133.1], *Pseudomonas* 23S rRNA [GenBank:Y00432.1], *CEACAM5* [GenBank:NM_004363.3], *CEACAM6* [GenBank:NM_002483.6], *CD74* [GenBank:NM_001025159.2], or *TMSB10* [GenBank:NM_021103.3]. The appropriate bacterial and human fragments were then placed directly next to each other, the reads supporting the integration were aligned to these adjacent fragments, and the insert-sizes were calculated for each read pair. The AD was then calculated by averaging the difference between the insert-size of the read pairs, *I*_*i*_ (*x*), and the median insert-size of the corresponding sequencing library, *M*_*SL*_. This process was repeated recursively by adding 1 ambiguous base between the bacterial and human fragments. By adding 0–100 bp bases between the two fragments, the number of bases, *x*, between the two fragments was titrated to yield the minimum AD. The one exception for this process was for participant D, as the bacterial reads wouldn’t align to these specific 16S and 23S rRNA references with the BWA default settings, despite OTUs parsimonious with a *Pseudomonas*-like bacterium. Instead, to maintain a consistent bacterial references, a default BLAST [28] search was performed to align the bacterial reads from participant D to these specific 16S and 23S rRNA references.

### Jensen-Shannon distance

For the JSD, the number of bases, *x*, was titrated between the bacterial and human fragments to yield the minimum JSD [29] for the insert-size distributions of the integration model (*d*_*I*_(*x*)) and the library (*d*_*SL*_) as implemented by Arumugam et al. [30, 31]. Briefly, a probability distribution was calculated for the insert-sizes (the number of reads with a given insert-size/total number of reads) of the model and reference data. A pseudocount of 0.0000001 was used to replace any insert-sizes with zero reads to avoid having zero values in either the nominator or denominator. Using these two distributions, the JSD was calculated between the model and library insert-size distributions. Bootstrap support for the model was calculated using 1000 iterations of calculating the confidence interval of the JSD. The JSD calculations were made with a custom Perl [32] script (Additional file 8: Text S1) that used the Statistics::R module [33], R [34], Bio::Perl [35], and samtools [26, 27]. To calibrate the JSD, paired-end reads from participant B’s library were randomly sampled using the Picard [25] DownsampleSam function.

### Cancer cell line analysis

Using the LGTSeek pipeline [13], two cancer cell line datasets were analyzed for evidence of bacterial DNA integrations into the human genome. One dataset (EGAD00001000725) was generated by Genentech, Inc and Genentech Research and Early Development [17] and is made available through the European Genome-Phenome Archive [36]. The other dataset was the Cancer Cell Line Encyclopedia [16] that was generated by a collaboration between the Broad Institute and Novartis Institutes for Biomedical Research and its Genomics Institute of the Novartis Research Foundation. The Cancer Cell Line Encyclopedia is publicly available through the University of California Santa Cruz Genomics Institute Cancer Genomics Hub [37].

### Validating the KPL-1 integration

The KPL-1 integration junction was PCR amplified with primers 5′-GGCTACCCGTGATATTGCTG-3′ and 5′-AGGTTTCAGCTGGTTTTTGC-3′ targeting the consensus bacterial and human fragments, respectively. The PCR was performed using Taq 2x Master Mix (New England BioLab, Ipswich, MA, USA) with 0.2 μM primer (Sigma-Aldrich, St. Louis, MO, USA) and 20 ng of KPL-1 genomic DNA (DSMZ, Braunschweig, Germany). Template was denatured at 95 **°**C for 60 s followed by 30 cycles of denaturing at 95 **°**C for 20 s, annealing at 60 **°**C for 30 s, and elongation at 68 **°**C for 40 s, followed by a final 5 min elongation at 68 **°**C. The resulting PCR product was then directly cloned into pCR 2.1-TOPO vector using the TOPO TA cloning kit following the manufacturer’s protocol followed by transformation into One Shot TOP10 chemically competent cells (Life Technologies, Grand Island, NY, USA). Plasmid was isolated from an overnight culture in LB with 0.1 μg/μL carbenicillin from a single isolated colony with the QIAprep Spin Miniprep kit (Qiagen, Valencia, CA, USA) following the manufacturer’s protocol. The plasmid was sequenced using standard M13 forward and reverse primers at the University of Maryland Institute for Genome Science Genome Resource Center and analyzed using CLC Genomics Workbench v.7.

### Guanine calculations

The %GC and %AG were calculated using custom perl scripts to the complete bacterial references for the genus *Pseudomonas* obtained from RefSeq (Additional file 9: Text S2), the 16S and 23S rRNA gene references for the genus *Pseudomonas* from the Silva database release 119 (http://www.arb-silva.de/), the hg38 human genome reference, and the hg38 transcriptome reference. The RPKM was calculated using custom perl scripts for the participants with bacterial DNA integrations. Transcripts with >0 RPKM were considered expressed. Differences between the %GC or %AG of the bacterial or human fragment consensus sequences and the references were tested using the Wilcoxon rank-sum test and Bonferroni corrected. The data are illustrated in Additional file 2: Figure S2 using R & the ggplot2 boxplot function. The outliers were not removed from any calculations. The expression data were stratified so that the lowest expressed transcripts are in Q1, while the highest expressed transcripts are in Q4.

### CpG islands and G-quartets

The non-B database [22] was searched for G-quartets in *CEACAM5*, *CEACAM6*, *CD74*, and *TMSB10.* The database did not report any identified motifs in the first exon of these genes using the search criteria “G Quadruplex Motif.”

The human genes with integrations were searched for CpG islands using: CpGProD [38], EMBOSS [39–41], and SMS [42]. Given that CpG islands are typically defined as ≥ 200 bp, the first exon of each gene and 1 kbp upstream of the TSS were searched for predicted CpG islands. The following references and coordinates for each gene were used for the CpG island searches: *CEACAM5* [GenBank:NC_000019.10, 41707611-41708795], *CEACAM6* [GenBank:NC_000019.10, 41754490-41755703], *CD74* [GenBank:NC_000005.10, 150412625-150413936], and *TMSB10* [GenBank:NC_000002.12, 84904639-84905718]. In order to analyze only the first exon for CpG islands, the EMBOSS algorithm settings were set so that the window size was 10 bp (default 100 bp) and a CpG island minimum length was 20 bp. The first exons were defined as: *CEACAM5* [GenBank:NC_000019.10, 41708611-41708795], *CEACAM6* [GenBank:NC_000019.10, 41755490-41755703], *CD74* [GenBank:NC_000005.10, 150412625-150412936], and *TMSB10* [GenBank:NC_000002.12, 84905639-84905718].

The average %GC content of the *Pseudomonas* genomes was calculated on all RefSeq *Pseudomonas* complete genomes available from RefSeq (release 01.05.2015). The %GC for the *Pseudomonas* 16S and 23S rRNA genes was calculated using all *Pseudomonas* rRNA genes from the SILVA rRNA database (release 119) [43]. The average %GC content of the human genome was calculated on all the nuclear chromosomes using hg19 and the RefSeq transcriptome (downloaded 04.01.15).

### Secondary structure

The *Pseudomonas* 16S [GenBank: M34133.1] and 23S [GenBank: Y00432.1] rRNA secondary structures were downloaded from the Comparative RNA Web Site and Project [44]. The human secondary structures of *CEACAM5, CEACAM6, CD74,* and *TMSB10* were predicted using the minimum free energy prediction by the RNAfold server [45, 46].

### Relative position of integrations

The absolute distance between the integration site and the TSS was calculated by taking the difference (bp) between the middle of each predicted site of integration and the TSS for the respective gene (Table 2, column 2). To calculate the relative position, the difference was divided by the total size of the first exon (bp) and multiplied by 100 to get the percent distance the integration is relative to the TSS and the end of the first exon (Table 2, column 3).

### Availability of data and materials

The datasets supporting the conclusions of this article are available in The Cancer Genome Atlas (TCGA) [phs000178, https://cghub.ucsc.edu] (Additional File 10: Table S1) , the Cancer Cell Line Encyclopedia (CCLE) [16] [CCLE, https://cghub.ucsc.edu], and Genentech Inc [17] [EGAD00001000725, https://www.ebi.ac.uk/ega/home]. Information about TCGA, the investigators, and the institutions who constitute the TCGA research network can be found at “http://cancergenome.nih.gov”. The TCGA data were obtained via the Sequence Read Archive as approved by dbGap. The CCLE data were generated by the Broad Institute and Novartis Institutes for Biomedical Research and its Genomics Institute of the Novartis Research Foundation Data. The CCLE data were made publicly available through the University of California Santa Cruz Genomics Institute CGHub. Information about the CCLE can be found at “https://cghub.ucsc.edu/datasets/ccle.html". The EGAD00001000725 data were generated by Genentech Inc and Genentech Research and Early Development. The University of Maryland, Baltimore, Institutional Review Board reviewed this study and determined that it did not require IRB review.

## Additional files

Additional file 1: Figure S1. Confirming the *CEACAM5* transcriptional start site annotation. The expression level around the TSS of *CEACAM5* is compared across all previously analyzed participants in the stomach adenocarcinoma and breast cancer data sets to that of the well-characterized *ACTB.* The *CEACAM5* expression is consistent with an accurate TSS annotation in the human genome. (PDF 3219 kb) Additional file 2: Figure S2. KPL1 breakpoint. An illustration of the junction of bacterial and human DNA from the KPL1 cell line. The breakpoint consists of bacterial DNA with homology to an aminoglycoside phosphotransferase gene, such as in the *Escherichia coli* K12. The human DNA is homologous to chromosome 6. There is a 24 bp bacterial inversion with overlap at the breakpoint. The bacterial and human DNA are 99 % (194/195 & 24/24) and 100 % (200/200) identical, respectively. (PDF 823 kb) Additional file 3: Figure S3. Guanine enrichment near bacterial DNA integrations. The distributions of the %GC and %AG (panels A & B, respectively) of the bacterial and human fragment consensus sequences were compared to other bacterial and human references. The expressed transcripts (>0 RPKM) in the participants with the bacterial DNA integrations were stratified based on RPKM into four quartiles (Q1 = red, Q2 = yellow, Q3 = green, Q4 = blue), with the lowest expressed transcripts in the first quartile (Q1) and the highest expressed transcripts in the fourth quartile (Q4). (PDF 158 kb) Additional file 4: Figure S4. EMBOSS CpG island search results. Using the EMBOSS CpG island prediction software, the *CEACAM5, CEACAM6, CD74, & TMSB10* bacterial DNA integrations were not found to be in proximity to CpG islands (A-F). The default EMBOSS algorithm was used to search the first exon and 1 kbp upstream of the TSS for each gene (A,C,E,&G), while altered parameters were used to search the first exon only (B,D,F,&H). (PDF 539 kb) Additional file 5: Figure S5. RNA secondary structure of the bacterial 16S & 23S integrated rRNA gene fragments. Each colored line indicates a fragment of the *Pseudomonas* 16S or 23S rRNA gene that is predicted to have integrated into the human genome. The lines are color-coded based on the participant and human gene the bacterial rRNA fragment that has integrated into (C5 = *CEACAM5*, C6 = *CEACAM6*, CD = *CD74*, T10 = *TMSB10*, “_1” are upstream rRNA fragments relative to the “_2” integrations). (PDF 2295 kb) Additional file 6: Figure S6. The predicted secondary structure of the human transcripts near the bacterial DNA integrations. The secondary structure for the examined human transcripts was predicted with the minimum free energy prediction from the RNAfold server (ViennaRNA v.2.2.0c) [45], and the location of the predicted integration of the *Pseudomonas* rRNA gene has been highlighted. (PDF 4110 kb) Additional file 7: Figure S7. Schematic of the paired-end reads color scheme. An illustration of the color scheme used to describe the relationship between the insert-size for the paired-end reads (i) and the median insert-size of a participant’s library. Lighter colors indicate the read pair’s insert-size is closer to the median library insert-size. For the purpose of this illustration, these hypothetical reads have a median insert-size of 200 bp and median absolute deviation (σ) of 20 bp. (PDF 171 kb) Additional file 8: Text S1. Code to calculate the models of DNA integration, AD & JSD. This code is a part of a custom Perl script that creates the models for an integration. Specifically, this code calculates the consensus sequence and the optimum distance between the two fragments using the average difference and Jensen-Shannon Distance. (PDF 53 kb) Additional file 9: Text S2.
*Pseudomonas* accessions. A list of GenBank accessions used to calculate the *Pseudomonas* genome %GC. (TXT 672 bytes) Additional file 10: Table S1 Conversion of the participant names to SRR accession number. (DOC 66 kb)

## Abbreviations

AD: Average difference
BRCA: Breast cancer
CCLE: Cancer Cell Line Encyclopedia
JSD: Jensen-Shannon distance
STAD: Stomach adenocarcinoma
TCGA: The Cancer Genome Atlas
TSS: Transcriptional start site
