# Supplementary figures and images for: Modeling the integration of bacterial rRNA fragments into the human cancer genome
Source: BMC Bioinformatics. 2016 Mar 21;17:134. doi: 10.1186/s12859-016-0982-0 (PMC4802584; doi:10.1186/s12859-016-0982-0)

# STAD

CEACAM5

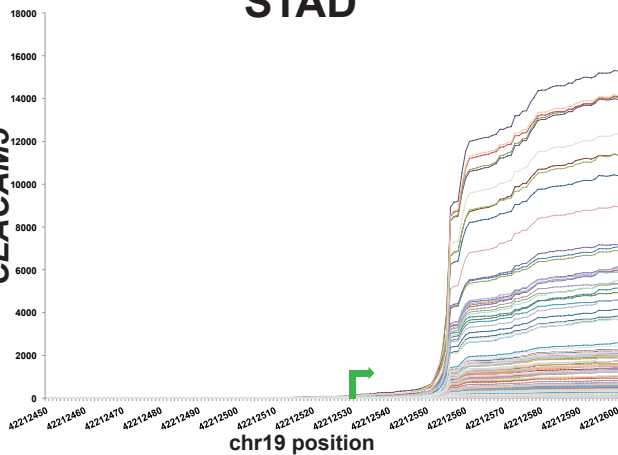

# BRCA

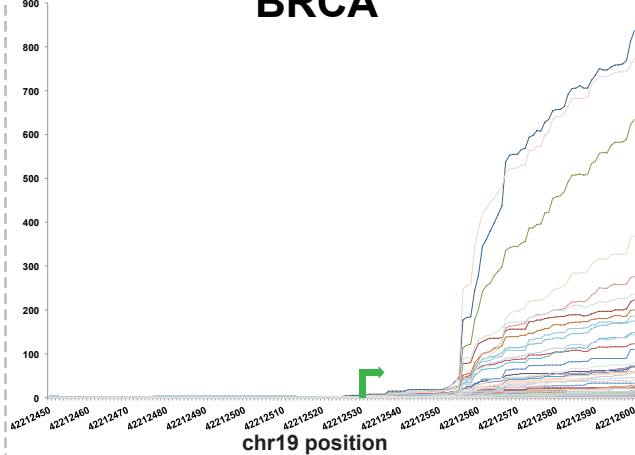

Beta-Actin

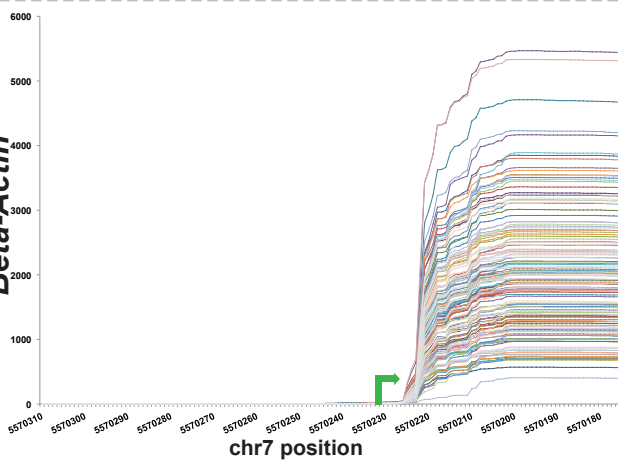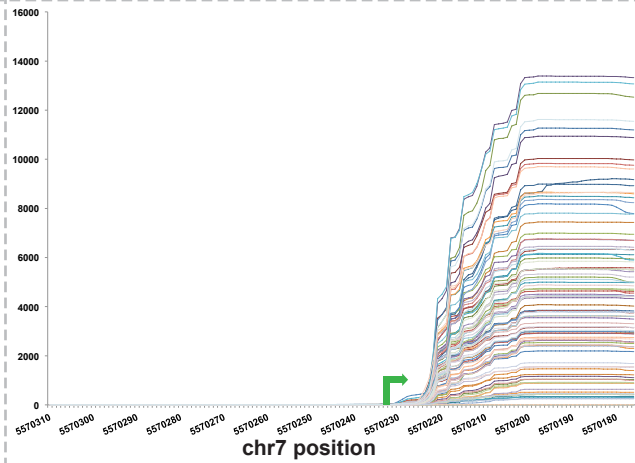

Supplement: Additional file 1: Figure S1. — Confirming the CEACAM5 transcriptional start site annotation. The expression level around the TSS of CEACAM5 is compared across all previously analyzed participants in the stomach adenocarcinoma and breast cancer data sets to that of the well-characterized ACTB. The CEACAM5 expression is consistent with an accurate TSS annotation in the human genome. (PDF 3219 kb) [file 12859_2016_982_MOESM1_ESM.pdf]

*Escherichia coli* K12  
GenBank: CP10439.1

KPL1

Human Chromosome 6  
GRCh38.p2

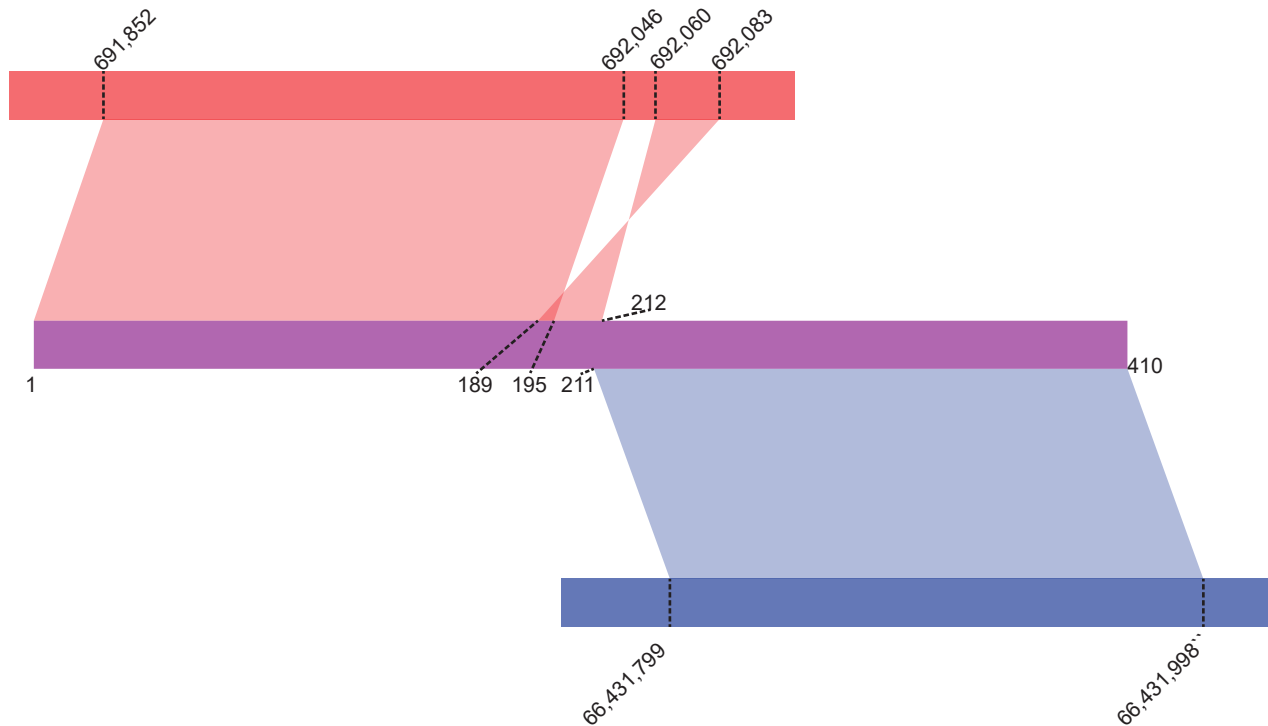

Supplement: Additional file 2: Figure S2. — KPL1 breakpoint. An illustration of the junction of bacterial and human DNA from the KPL1 cell line. The breakpoint consists of bacterial DNA with homology to an aminoglycoside phosphotransferase gene, such as in the Escherichia coli K12. The human DNA is homologous to chromosome 6. There is a 24 bp bacterial inversion with overlap at the breakpoint. The bacterial and human DNA are 99 % (194/195 & 24/24) and 100 % (200/200) identical, respectively. (PDF 823 kb) [file 12859_2016_982_MOESM2_ESM.pdf]

**A**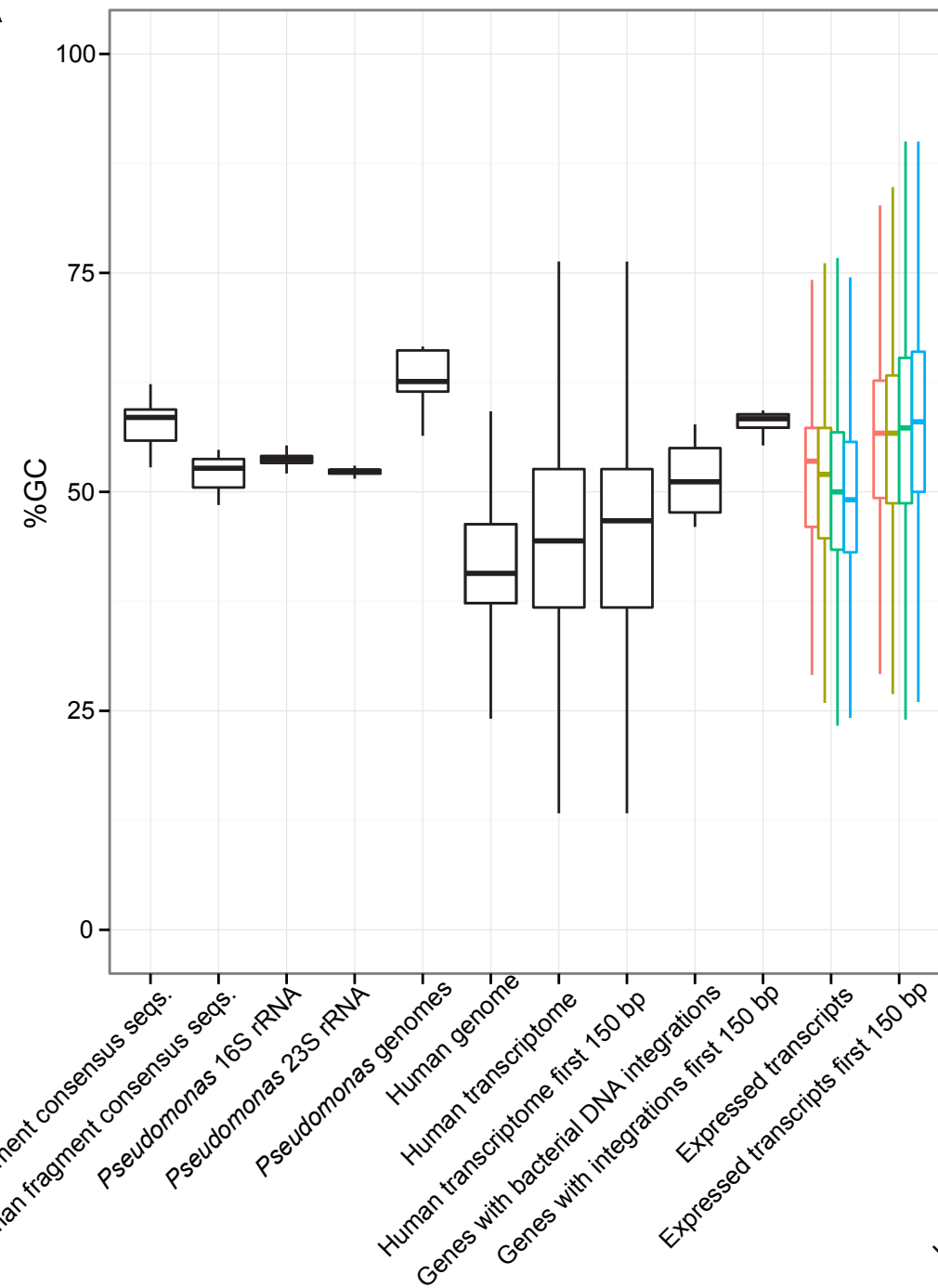**B**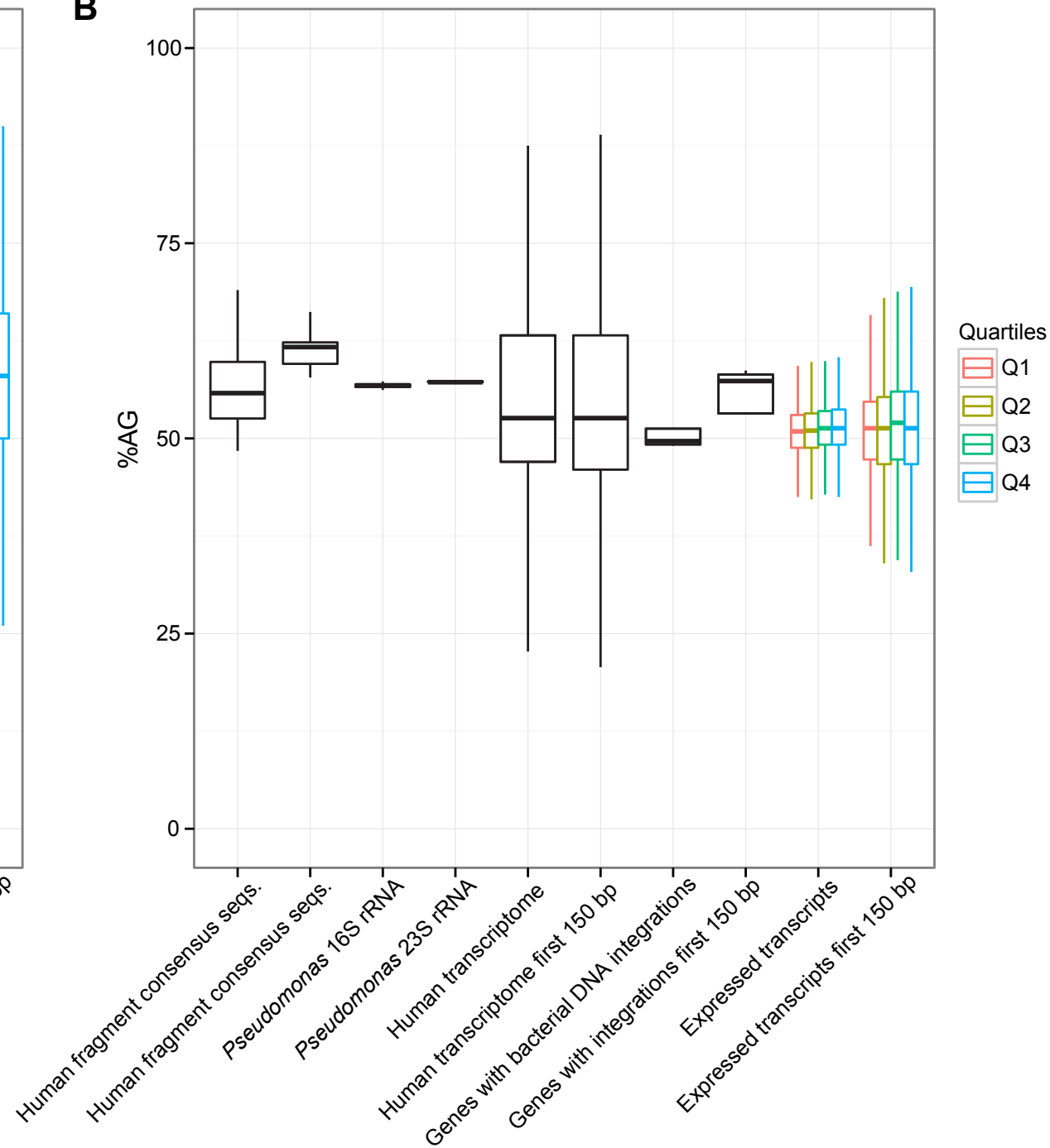

Supplement: Additional file 3: Figure S3. — Guanine enrichment near bacterial DNA integrations. The distributions of the %GC and %AG (panels A & B, respectively) of the bacterial and human fragment consensus sequences were compared to other bacterial and human references. The expressed transcripts (>0 RPKM) in the participants with the bacterial DNA integrations were stratified based on RPKM into four quartiles (Q1 = red, Q2 = yellow, Q3 = green, Q4 = blue), with the lowest expressed transcripts in the first quartile (Q1) and the highest expressed transcripts in the fourth quartile (Q4). (PDF 158 kb) [file 12859_2016_982_MOESM3_ESM.pdf]

# 1 kb promoter & Exon1

# Exon1

A.

CEACAM5

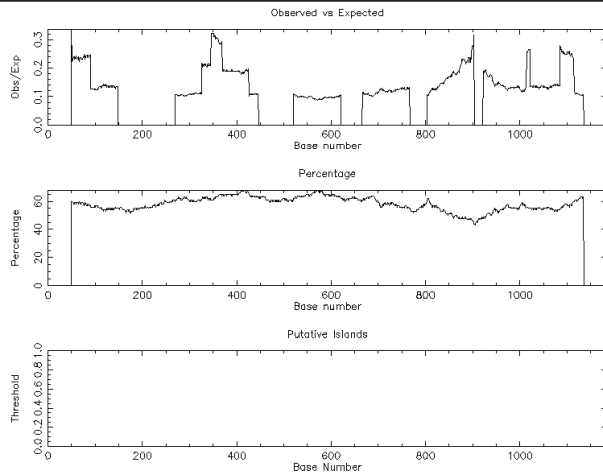

B.

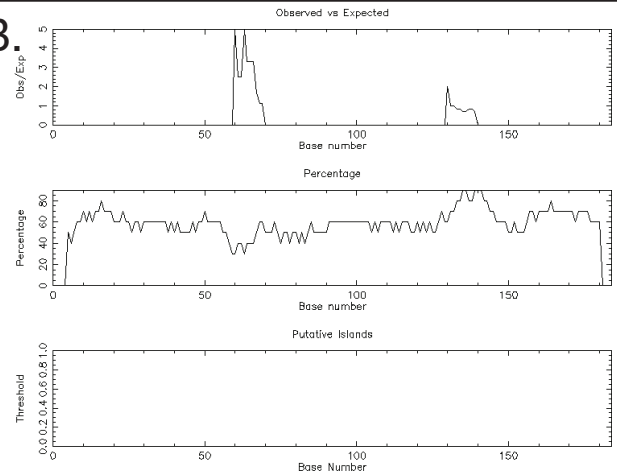

C.

CEACAM6

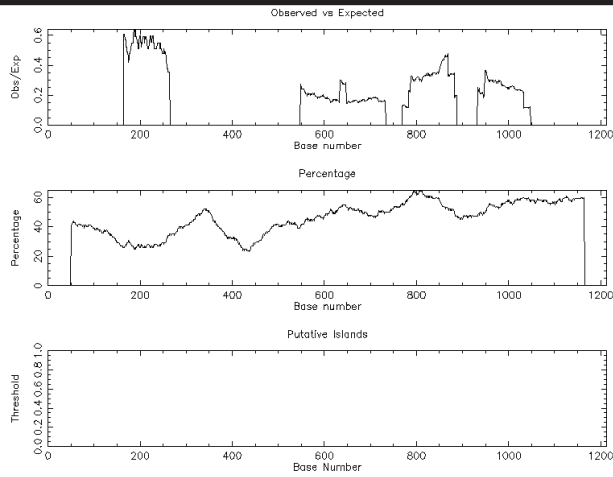

D.

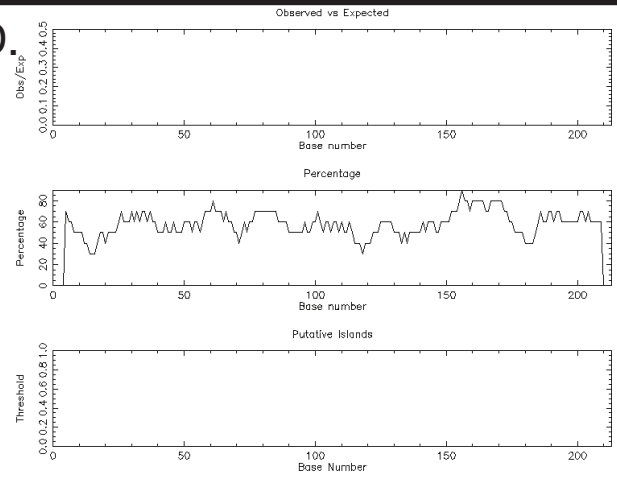

E.

CD74

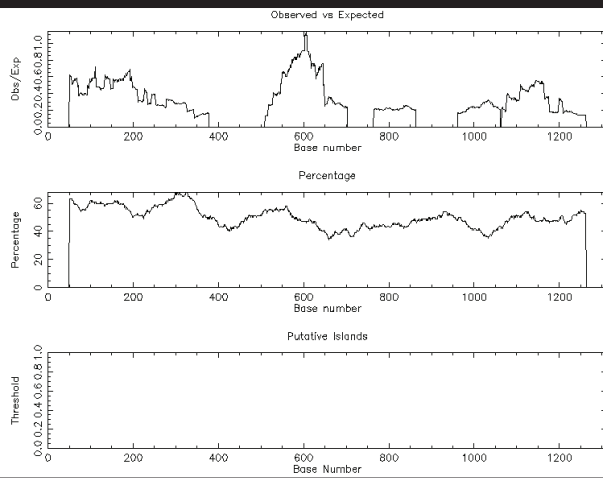

F.

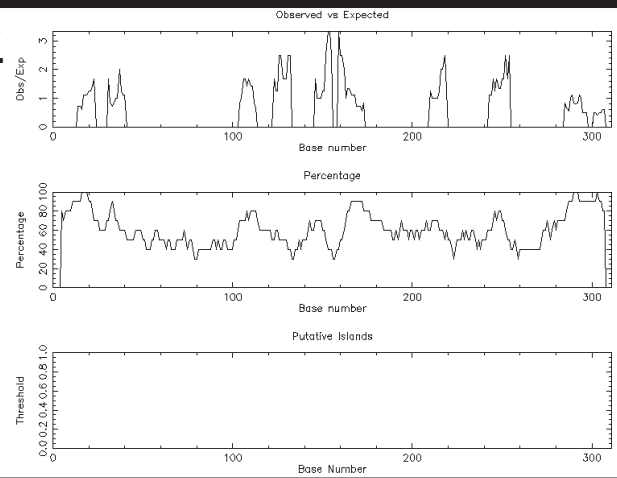

G.

TMSB10

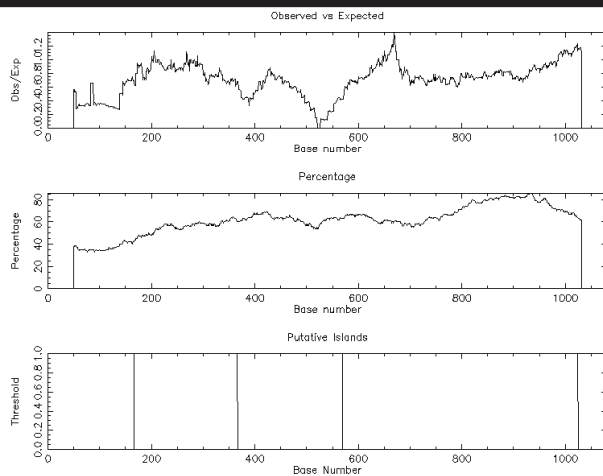

H.

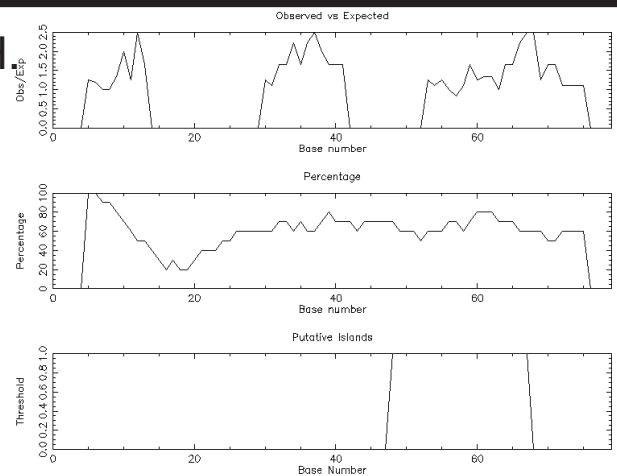

Supplement: Additional file 4: Figure S4. — EMBOSS CpG island search results. Using the EMBOSS CpG island prediction software, the CEACAM5, CEACAM6, CD74, & TMSB10 bacterial DNA integrations were not found to be in proximity to CpG islands (A-F). The default EMBOSS algorithm was used to search the first exon and 1 kbp upstream of the TSS for each gene (A,C,E,&G), while altered parameters were used to search the first exon only (B,D,F,&H). (PDF 539 kb) [file 12859_2016_982_MOESM4_ESM.pdf]

# Distance from the median insert

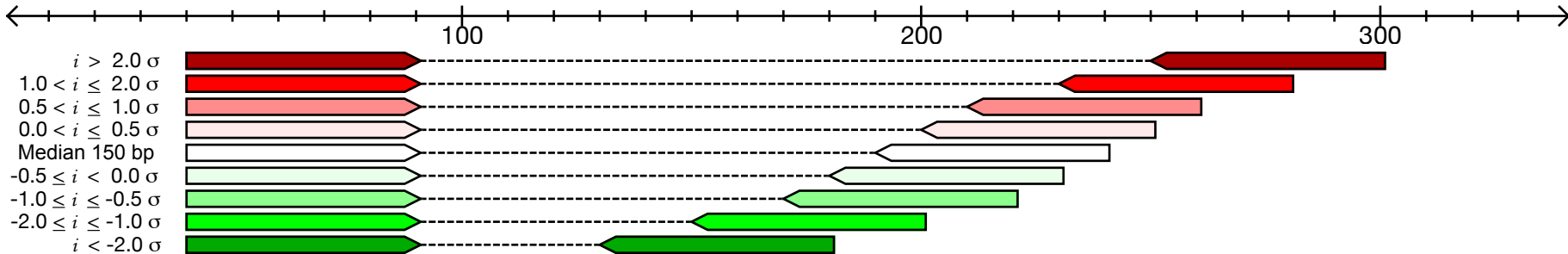

Supplement: Additional file 7: Figure S7. — Schematic of the paired-end reads color scheme. An illustration of the color scheme used to describe the relationship between the insert-size for the paired-end reads (i) and the median insert-size of a participant’s library. Lighter colors indicate the read pair’s insert-size is closer to the median library insert-size. For the purpose of this illustration, these hypothetical reads have a median insert-size of 200 bp and median absolute deviation (σ) of 20 bp. (PDF 171 kb) [file 12859_2016_982_MOESM7_ESM.pdf]
