# Supplementary material for: Modeling the integration of bacterial rRNA fragments into the human cancer genome
Source: BMC Bioinformatics. 2016 Mar 21;17:134. doi: 10.1186/s12859-016-0982-0 (PMC4802584; doi:10.1186/s12859-016-0982-0)
Supplement: Additional file 5: Figure S5. — RNA secondary structure of the bacterial 16S & 23S integrated rRNA gene fragments. Each colored line indicates a fragment of the Pseudomonas 16S or 23S rRNA gene that is predicted to have integrated into the human genome. The lines are color-coded based on the participant and human gene the bacterial rRNA fragment that has integrated into (C5 = CEACAM5, C6 = CEACAM6, CD = CD74, T10 = TMSB10, “_1” are upstream rRNA fragments relative to the “_2” integrations). (PDF 2295 kb) [file 12859_2016_982_MOESM5_ESM.pdf]

Pseudomonas aeruginosa (M34133)
Secondary Structure: small subunit ribosomal RNA

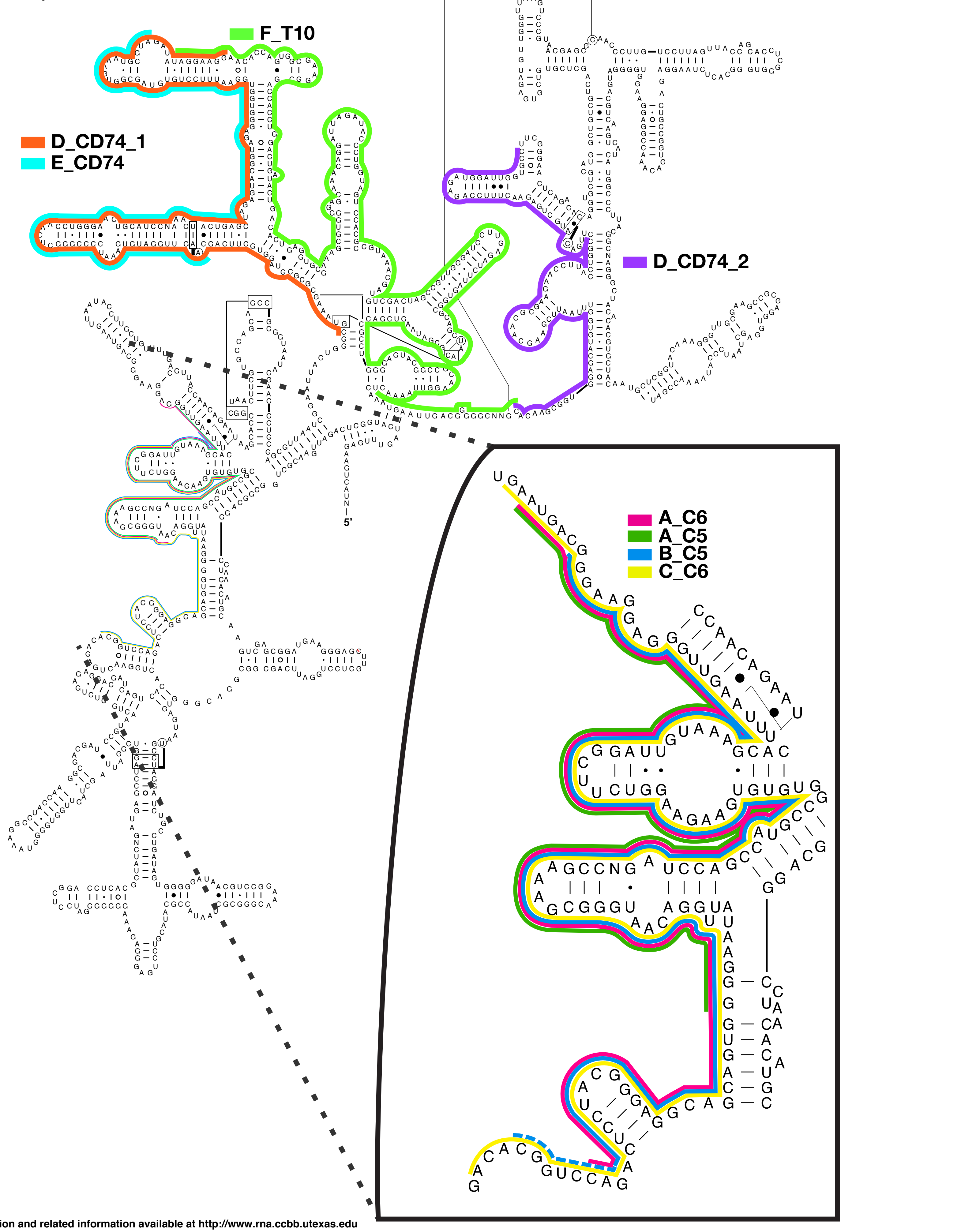

Citation and related information available at http://www.rna.ccbb.utexas.edu

Secondary Structure: large subunit ribosomal RNA - 5' half

Secondary Structure: large subunit ribosomal RNA - 3' half

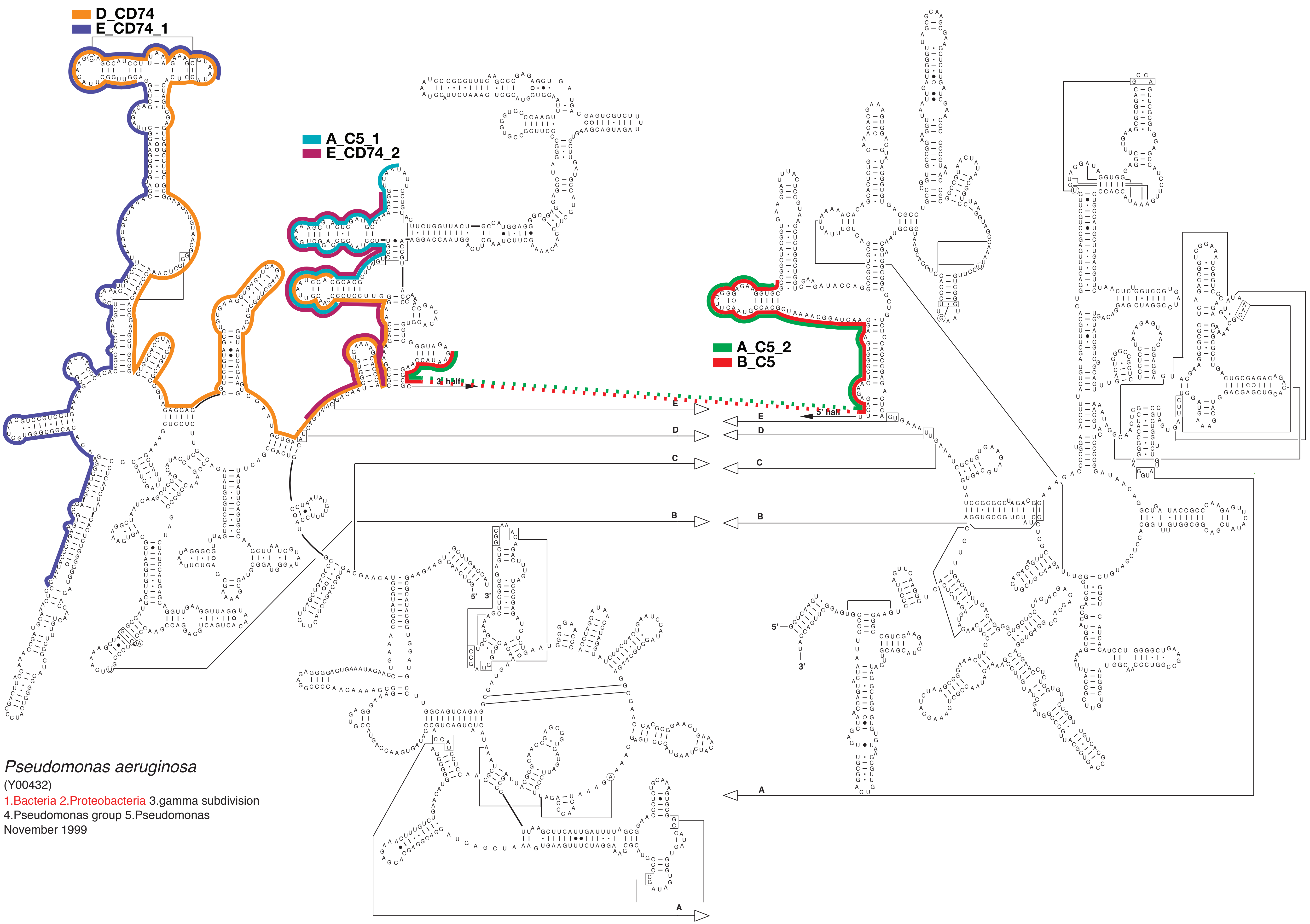

Pseudomonas aeruginosa

(Y00432)

1.Bacteria 2.Proteobacteria 3.gamma subdivision

4.Pseudomonas group 5.Pseudomonas

November 1999

Citation and related information available at http://www.rna.icmb.utexas.edu
