# Supplementary material for: Modeling the integration of bacterial rRNA fragments into the human cancer genome
Source: BMC Bioinformatics. 2016 Mar 21;17:134. doi: 10.1186/s12859-016-0982-0 (PMC4802584; doi:10.1186/s12859-016-0982-0)
Supplement: Additional file 6: Figure S6. — The predicted secondary structure of the human transcripts near the bacterial DNA integrations. The secondary structure for the examined human transcripts was predicted with the minimum free energy prediction from the RNAfold server (ViennaRNA v.2.2.0c) [45], and the location of the predicted integration of the Pseudomonas rRNA gene has been highlighted. (PDF 4110 kb) [file 12859_2016_982_MOESM6_ESM.pdf]

Predicted *CEACAM5* mRNA Secondary Structure with predicted bacterial DNA integration regions overlayed.

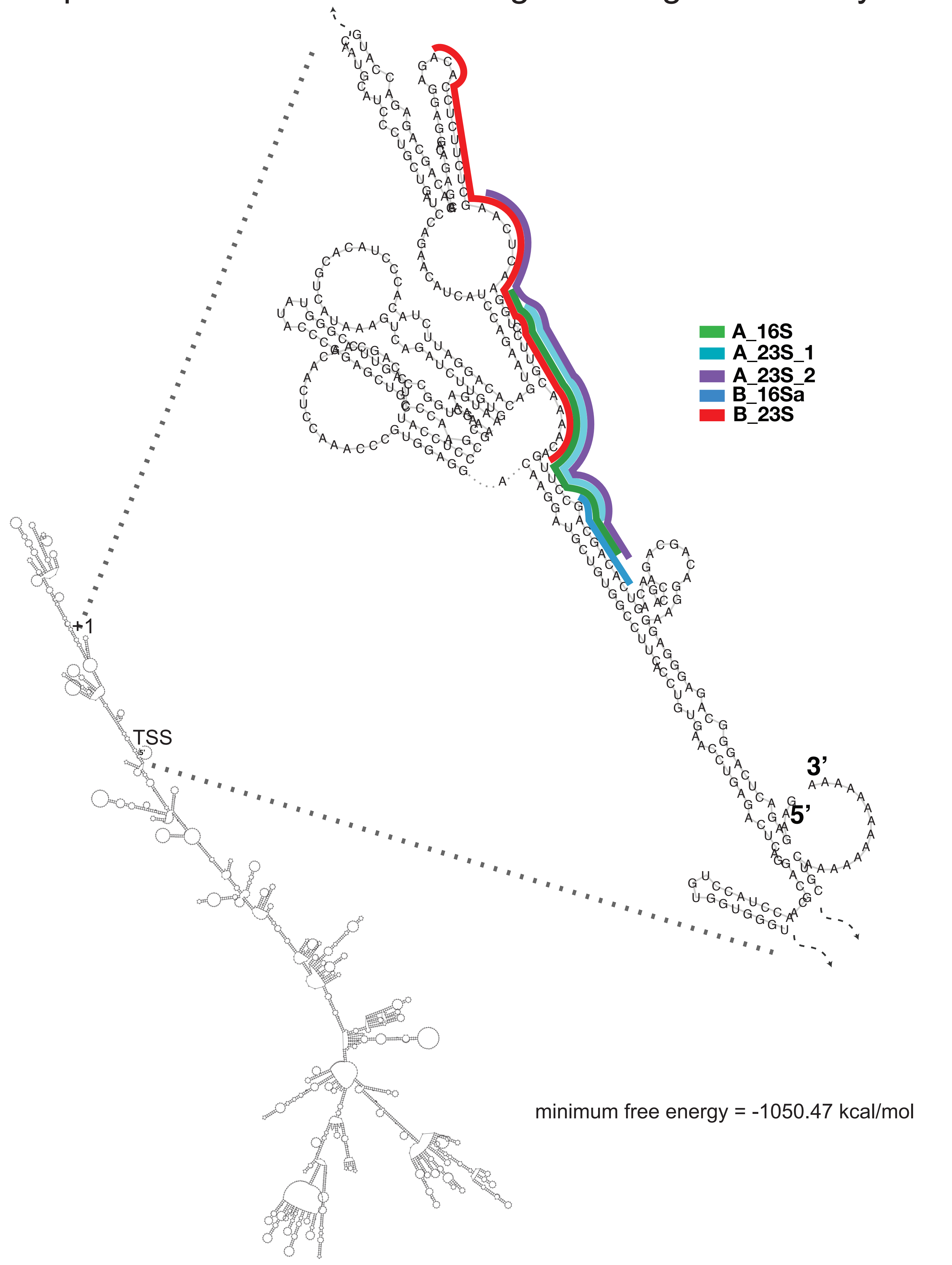

Predicted *CEACAM6* mRNA Secondary Structure with predicted bacterial DNA integration regions overlayed.

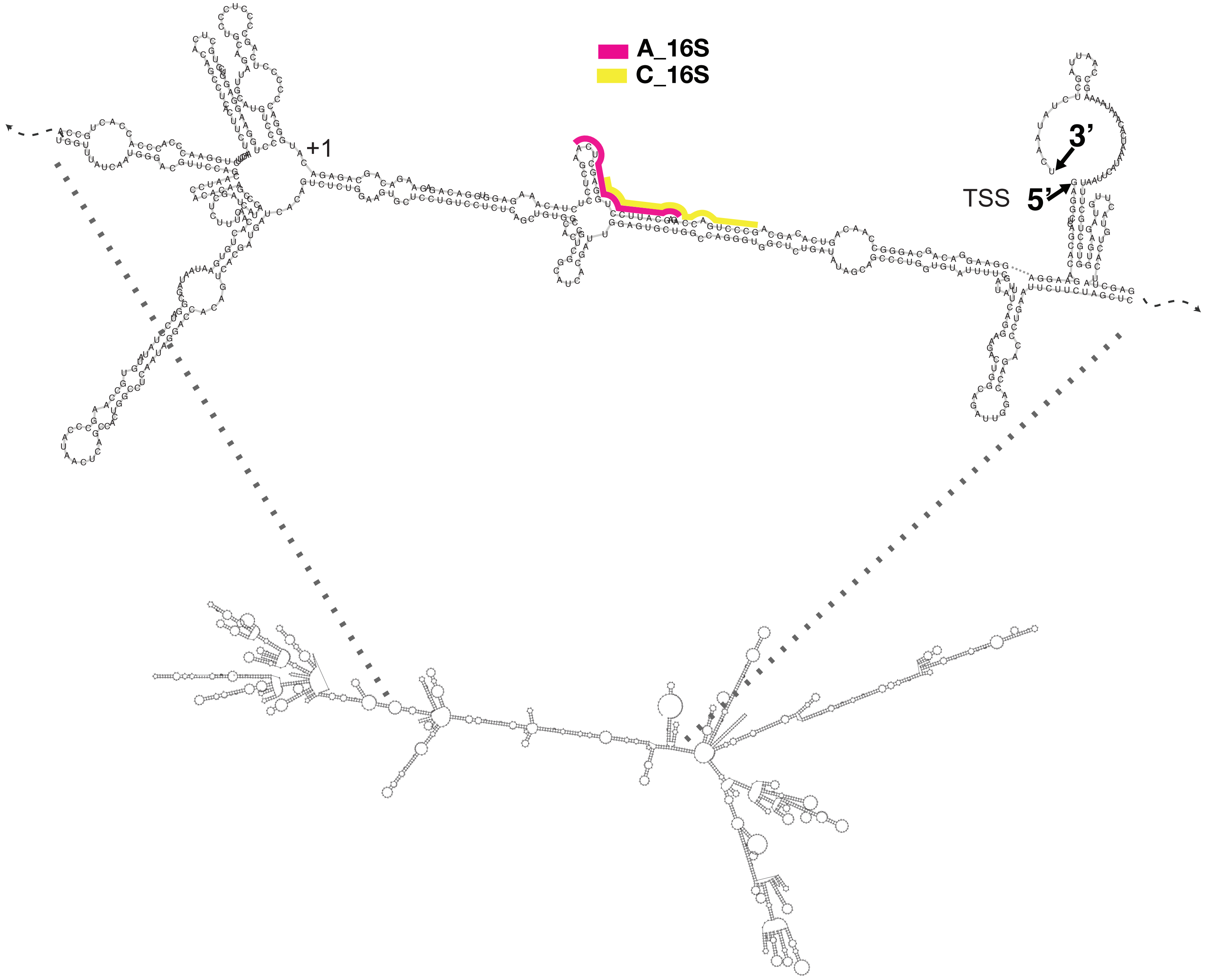

minimum free energy = -698.30 kcal/mol

Predicted *CD74* mRNA Secondary Structure with predicted bacterial DNA integration regions overlayed.

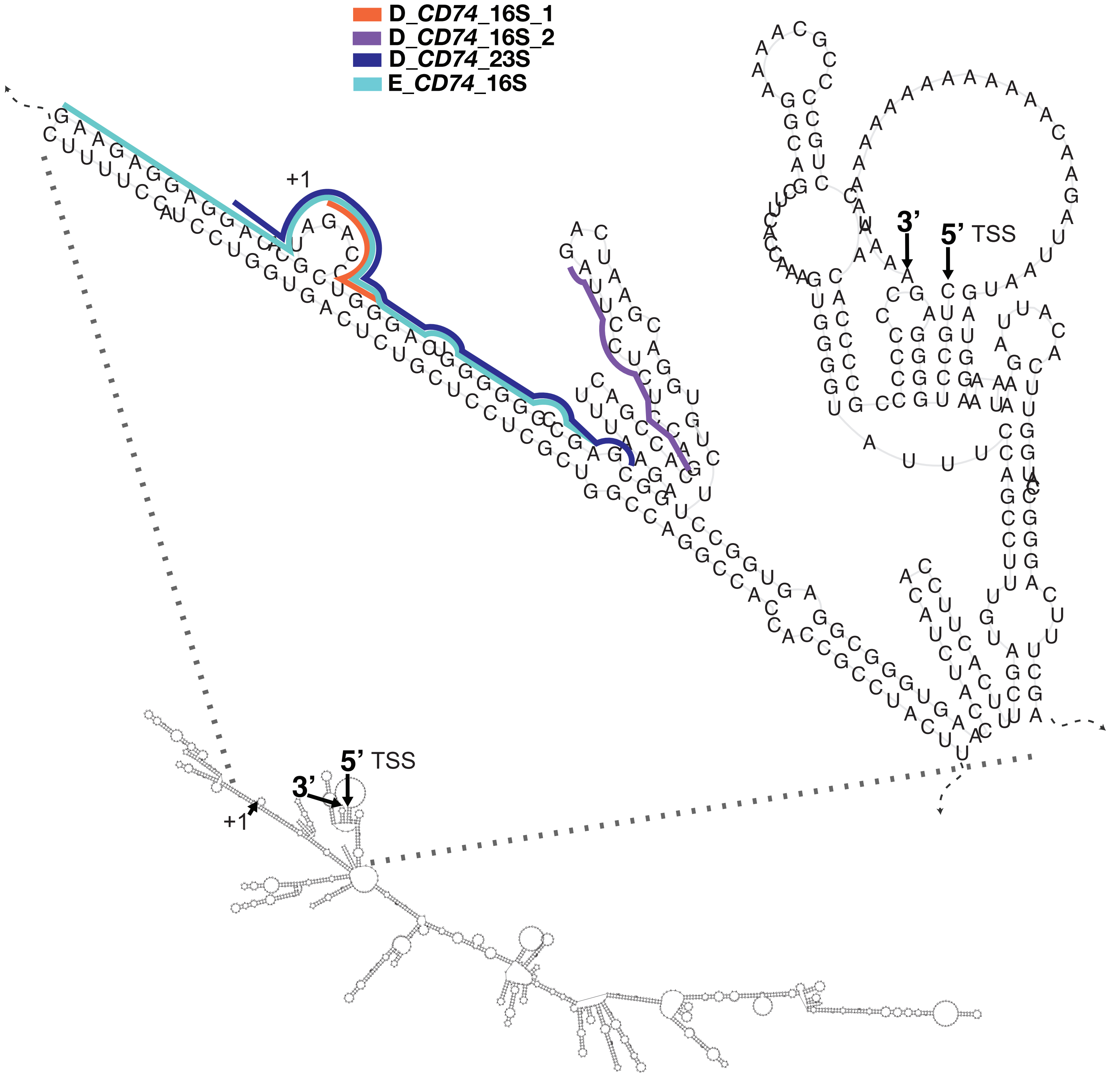

minimum free energy = -601.70 kcal/mol

Predicted *TMSB10* mRNA secondary structure with predicted bacterial DNA integration region overlayed.

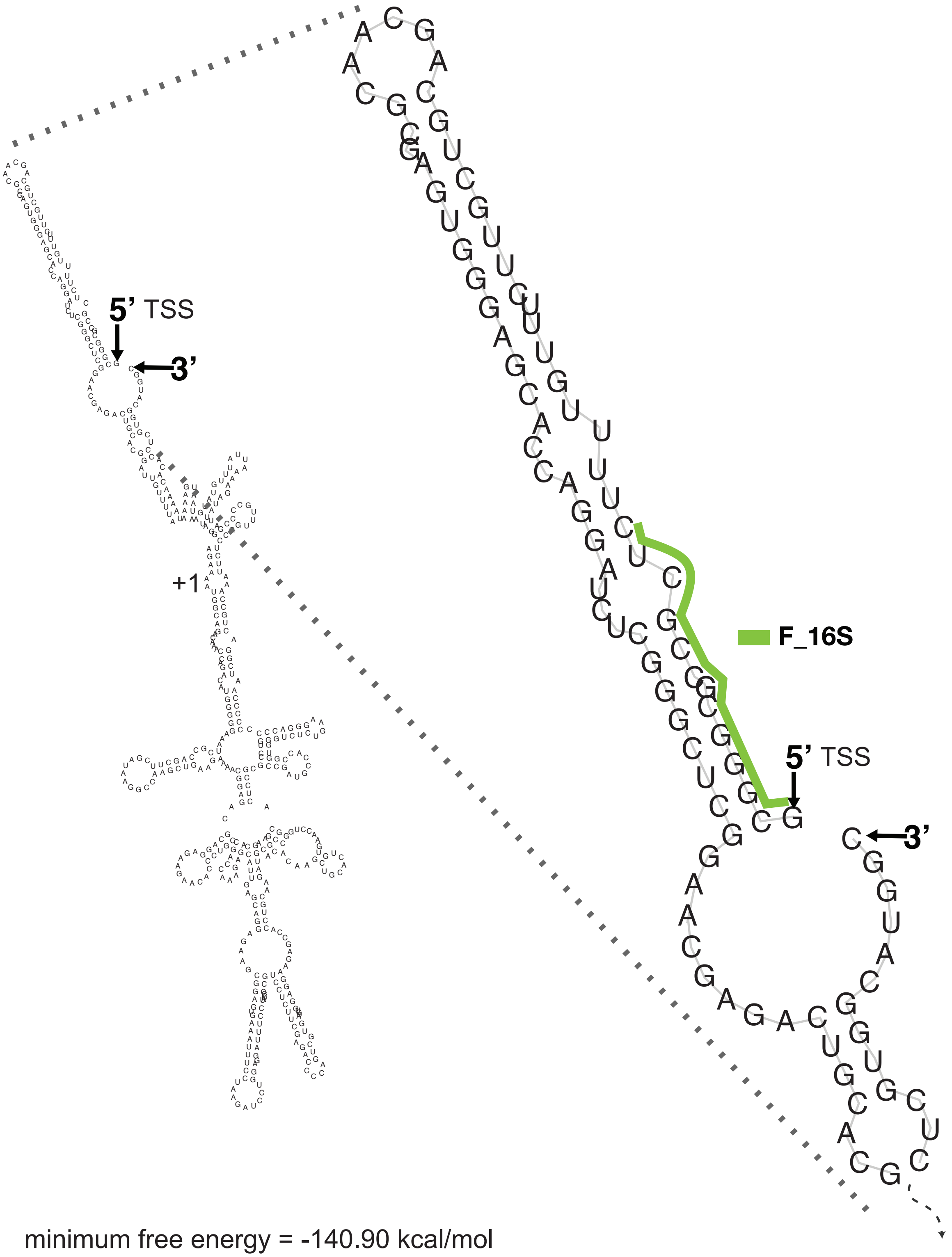

minimum free energy = -140.90 kcal/mol
