# Supplementary material for: Modeling the integration of bacterial rRNA fragments into the human cancer genome
Source: BMC Bioinformatics. 2016 Mar 21;17:134. doi: 10.1186/s12859-016-0982-0 (PMC4802584; doi:10.1186/s12859-016-0982-0)
Supplement: Additional file 8: Text S1. — Code to calculate the models of DNA integration, AD & JSD. This code is a part of a custom Perl script that creates the models for an integration. Specifically, this code calculates the consensus sequence and the optimum distance between the two fragments using the average difference and Jensen-Shannon Distance. (PDF 53 kb) [file 12859_2016_982_MOESM8_ESM.pdf]

```
=head1

Title   : &optimize_refs
Function: Calculate the ideal distance between the two integration (INT) references (refs) based on insert size (i_size).
Returns : A list of reference positions and a # of bp between both references to draw to illustrate the optimized INT configuration.
Usage   : my ( $ref1_data_list, $ref2_data_list, @n_num_list ) = &optimize_refs(
            {   bam1_data         => $bam1_data,
                bam2_data         => $bam2_data,
                merged_bam        => $merged_bam,
                ref1              => $ref1,
                ref2              => $ref2,
                picard_file       => /path/to/LIB_picard_insert_size_metrics.txt
                output_dir        => /path/for/output/
            });

Args    :
            bam1_data         => $bam1_data             ( &pull_bam_data object )
            bam2_data         => $bam2_data             ( &pull_bam_data object )
            merged_bam        => $merged_bam            ( &merge_Bams object )
            ref1              => /path/to/ref_1.fa
            ref2              => /path/to/ref_2.fa
            picard_file       => /path/to/picard_insert_size_metrics.txt for LIB
            output_dir        => /path/for/output,
            MM_only           => <0|1> 1= Only use reads pairs that have both pairs map to the merged refernce
            jsd               => <0|1> 1= Use Jensen-Shannon Distance calculations to determine distance between the 2 INT refs.
            titrate_n_string  => <0|1> 1= Titrated the LIB_stdev distances between the 2 refs for visualizing opti distance.
            insert_size       => LIB insert size    (overrides picard file parsing)
            stdev             => LIB stdev          (overrides picard file parsing)

Workflow:
            1. Init LIB i_size counts
            2. Init INT i_size counts when INT refs are adjacent
                2A. Create a reference with zero bases between ( "N_0" ) the two sides of the integration refs
                2B. Map merged.bam @ N_0 ref we created (2A). Calc INT i_sizes.
                2C. Init INT i_size counts from the picard file (2B).
            3. Titrate the optimal distance between the two references using JSD and AD
            4. Return a list of the ref sequences and opti-N

Input object structure:
        pull_bam_data object:
            'file'              => /file/path/input.bam
            'id_hash'           => \%bam_data_hash,             ## $bam_data->{id_hash}->{$id}=$corresponding_bam_line_data
            'header'            => \@samtools_header
            'strand'            => Integer. Positive means more reads map to + strand.
            'bam_region'        => 'chr:100-200'
            'rvcmplt'           => <0|1>
```

```
        merge_Bams object:
            'file'              => /path/to/file.bam,
            'ids'               => $hash{$ids}
            'count'             => number of reads,
            'bam1_strand'       => #, > 0 = more reads map positive strand, < 0 more reads map to the reverse strand
            'bam2_strand'       => #

=cut

sub optimize_refs {
    my $opts = shift;
    if (   !$opts->{bam1_data}
        || !$opts->{bam2_data}
        || !$opts->{ref2}
        || !$opts->{ref1}
        || !$opts->{merged_bam} )
    {
        confess "Error: Must pass &optimize_refs the following opts: bam1_data, bam2_data, ref1, ref2, merged_ids\n";
    }

    # Local variables
    my $bam1_data           = $opts->{bam1_data};
    my $bam2_data           = $opts->{bam2_data};
    my $merged_bam          = $opts->{merged_bam};
    my $MM_only             = defined $opts->{MM_only} ? $opts->{MM_only} : "1";
    my $jsd                 = defined $opts->{jsd} ? "$opts->{jsd}" : "0";
    my $tmp_optimal_ref_dir = "$opts->{output_dir}/tmp_optimal_ref_dir/";
    mk_dir($tmp_optimal_ref_dir);

    # Data to return
    my @ret_ref1_data_list;
    my @ret_ref2_data_list;
    my @n_num_list;

    # 1. Initialize count of the LIB i_size population
    ## This is based on the picard file for the LIB mapped at the appropriate reference
    my %LIB_count;
    my $LIB_stdev;
    my $LIB_median_insert_size;
    open( PIC, "<", "$opts->{picard_file}" )
        or confess "Error: Unable to open picard_file for reading: $opts->{picard_file}\n";
    my $header_1 = 1;
    while (<PIC>) {
        chomp( my $picard_data_line = $_ );
        if ( $picard_data_line =~ /^MEDIAN_INSERT_SIZE/ ) {
```

```
            chomp( my $picard_insert_sizes = <PIC> );
            my ( $fr_median_i_size, $fr_abs_deviation, $fr_mean_INT_i_sizes, $fr_stdev ) = ( split /\t/, $picard_insert_sizes )[ 0, 1, 4, 5 ];
            $LIB_stdev              = defined $opts->{stdev}       ? $opts->{stdev}       : $fr_abs_deviation;
            $LIB_median_insert_size = defined $opts->{insert_size} ? $opts->{insert_size} : $fr_median_i_size;
        }
        elsif ( $picard_data_line =~ /^insert_size/ ) { $header_1 = 0; next; }
        elsif ( $header_1 == 1 )                      { next; }
        elsif ( $picard_data_line =~ /^\d+/ ) {
            my ( $i_size, $fr, $rf, $tandem ) = split( /\t/, $_ );
            $LIB_count{$i_size} = $fr;
        }
    }
    close PIC;

    # 2. Initilize a count of the INT insert-size population with 0 bp between the two references ( N_0 )
    ## 2A First, make a reference with adjacent consensus sequences for ref1 & ref2

    # 2A.1 Create the consensus for the reads for each bam and respective bam_region
    print STDERR "======== Calculating the consensus sequences for each side of the INT ========\n";
    ## Bam1
    my $half_threads = floor( $threads / 2 );
    open( my $OUT_1_vcf_fh,
        "|-", "samtools view - -u | samtools sort -@ $half_threads -O bam -T tmp_bam1_sort - | samtools mpileup -uAf $opts->{ref1} - | bcftools
call -m -O z > $tmp_optimal_ref_dir/bam1_ref1.vcf.gz" )
        or die "Error: Unable to open a filehandle_1 to the VCF command.\n";
    print $OUT_1_vcf_fh @{ $bam1_data->{header} };

    ## Bam2
    open( my $OUT_2_vcf_fh,
        "|-", "samtools view - -u | samtools sort -@ $half_threads -O bam -T tmp_bam2_sort - | samtools mpileup -uAf $opts->{ref2} - | bcftools
call -m -O z > $tmp_optimal_ref_dir/bam2_ref2.vcf.gz" )
        or die "Error: Unable to open a filehandle_2 to the VCF command.\n";
    print $OUT_2_vcf_fh @{ $bam2_data->{header} };

    # Local variables to capture position data of the reads from the merged data.
    ## Some reads may be removed between regions_of_coverage (not INT specific) to merged_bam (INT specific).
    ## Losing reads may have altered the exact region of the INT so we recalculate it here.
    ## Bam1
    my $bam1_chr;
    my $bam1_min;
    my $bam1_max;
    ## Bam2
    my $bam2_chr;
    my $bam2_min;
    my $bam2_max;
```

```
# Print the bam data foreach read to the VCF while capturing position data
foreach my $read_id ( keys %{ $merged_bam->{ids} } ) {
    my $bam1_line = $bam1_data->{id_hash}->{$read_id};
    my $bam2_line = $bam2_data->{id_hash}->{$read_id};

    print $OUT_1_vcf_fh "$bam1_line\n";
    print $OUT_2_vcf_fh "$bam2_line\n";

    my @bam1_split = split( /\t/, $bam1_line );
    my @bam2_split = split( /\t/, $bam2_line );

    if ( !$bam1_chr ) { $bam1_chr = $bam1_split[2]; }
    if ( !$bam2_chr ) { $bam2_chr = $bam2_split[2]; }

    my $bam1_read_id_5position = $bam1_split[3];
    my $bam2_read_id_5position = $bam2_split[3];

    my $bam1_read_id_3position = $bam1_split[3] + length( $bam1_split[9] ) - 1;    ## Subtract 1 b/c of zero based counting
    my $bam2_read_id_3position = $bam2_split[3] + length( $bam2_split[9] ) - 1;    ## Subtract 1 b/c of zero based counting

    if ( !$bam1_min ) { $bam1_min = $bam1_read_id_5position; }
    if ( !$bam2_min ) { $bam2_min = $bam2_read_id_5position; }

    if ( !$bam1_max ) { $bam1_max = $bam1_read_id_3position; }
    if ( !$bam2_max ) { $bam2_max = $bam2_read_id_3position; }

    if ( $bam1_read_id_5position < $bam1_min ) { $bam1_min = $bam1_read_id_5position; }
    if ( $bam2_read_id_5position < $bam2_min ) { $bam2_min = $bam2_read_id_5position; }

    if ( $bam1_read_id_3position >= $bam1_max ) {
        $bam1_max = $bam1_read_id_3position;
    }
    if ( $bam2_read_id_3position >= $bam2_max ) {
        $bam2_max = $bam2_read_id_3position;
    }
}

close $OUT_1_vcf_fh;
close $OUT_2_vcf_fh;

# Index the VCF file
run_cmd("tabix $tmp_optimal_ref_dir/bam1_ref1.vcf.gz");
run_cmd("tabix $tmp_optimal_ref_dir/bam2_ref2.vcf.gz");

# Create the consensus sequences
```

```
    run_cmd("samtools faidx $opts->{ref1} \'$bam1_chr\:$bam1_min\-$bam1_max\' | bcftools consensus $tmp_optimal_ref_dir/bam1_ref1.vcf.gz >
$tmp_optimal_ref_dir/bam1_ref1.fa");
    run_cmd("samtools faidx $opts->{ref2} \'$bam2_chr\:$bam2_min\-$bam2_max\' | bcftools consensus $tmp_optimal_ref_dir/bam2_ref2.vcf.gz >
$tmp_optimal_ref_dir/bam2_ref2.fa");

    # Determine if we need to flip the orientation of the consensus sequence in order to have INT reads facing eachother. If we have to flip it,
make an output-note of it.
    ## Bam1
    my $bam1_region_0 = ( $bam1_data->{rvcmplt} == 1 ) ? "$bam1_chr\:$bam1_max\-$bam1_min" : "$bam1_chr\:$bam1_min\-$bam1_max";
    if ( $bam1_data->{rvcmplt} == 1 ) {
        open( OUT, ">", "$opts->{output_dir}/REVERSE_COMPLEMENTED_$bam1_region_0.txt" )
            or confess "Error: Unable to open output: $opts->{output_dir}/REVERSE_COMPLEMENTED.txt\n";
        print OUT "REVERSE_COMPLEMENTED: $bam1_region_0\n";
        close OUT;
    }
    ## Bam2
    my $bam2_region_0 = ( $bam2_data->{rvcmplt} == 1 ) ? "$bam2_chr\:$bam2_max\-$bam2_min" : "$bam2_chr\:$bam2_min\-$bam2_max";
    if ( $bam2_data->{rvcmplt} == 1 ) {
        open( OUT, ">", "$opts->{output_dir}/REVERSE_COMPLEMENTED_$bam2_region_0.txt" )
            or confess "Error: Unable to open output: $opts->{output_dir}/REVERSE_COMPLEMENTED.txt\n";
        print OUT "REVERSE_COMPLEMENTED: $bam2_region_0\n";
        close OUT;
    }

    # Open Bio::SeqIO to parse the seq to create the adjacent reference
    ## Bam1
    my $consensus1_fh      = Bio::SeqIO->new( -format => 'Fasta', -file => "$tmp_optimal_ref_dir/bam1_ref1.fa" );
    my $ref1_consensus     = $consensus1_fh->next_seq();
    my $ref1_consensus_seq = ( $bam1_data->{rvcmplt} == 1 ) ? $ref1_consensus->revcom()->seq() : $ref1_consensus->seq();
    ## Bam2
    my $consensus2_fh      = Bio::SeqIO->new( -format => 'Fasta', -file => "$tmp_optimal_ref_dir/bam2_ref2.fa" );
    my $ref2_consensus     = $consensus2_fh->next_seq();
    my $ref2_consensus_seq = ( $bam2_data->{rvcmplt} == 1 ) ? $ref2_consensus->revcom()->seq() : $ref2_consensus->seq();

    # Now that we have the sequence and region for both INT references, add them to the list of data to return so that we can draw it later
    push(
        @ret_ref1_data_list,
        {   'seq'   => $ref1_consensus_seq,
            'range' => $bam1_region_0,
        }
    );
    push(
        @ret_ref2_data_list,
        {   'seq'   => $ref2_consensus_seq,
            'range' => $bam2_region_0,
        }
```

```
);
push( @n_num_list, "0" );

# Create the adjacent reference for the INT with N=0. This will allow us to accurately calculate the i_size for the INT with N=0.
print STDERR "======== Create a new reference with both side of the INT adjacent to eachother =========\n";
my $adjacent_model_fa = "$tmp_optimal_ref_dir/adjacent_model_refs.fa";
open( my $REF, ">", $adjacent_model_fa ) or die "Error: &optimize_refs unable to open output model reference: $adjacent_model_fa\n";
## Print fasta header
print $REF ">adjacent_model_ref::$bam1_region_0\__$bam2_region_0\n";
## Print fasta sequence
print $REF $ref1_consensus_seq . $ref2_consensus_seq . "\n";
close $REF;
$consensus1_fh->close();
$consensus2_fh->close();

# 2B. Map the merged bam at the merged_N0_reference
print STDERR "======== BWA aln INT reads to the N_0 INT-Ref =========\n";
## bwa index merged_N0_reference
run_cmd("bwa index $adjacent_model_fa");
## bwa align & use Picard to calculate the i_size for the merged INT reads
my $adjacent_model_refs_bam = &bwa_aln(
    $opts->{merged_bam}->{file},
    $adjacent_model_fa,
    {   output_prefix  => "adjacent_model_refs",
        output_dir     => $tmp_optimal_ref_dir,
        insert_metrics => 1,
        MM_only        => $MM_only,
        cmd_log        => 1
    }
);

# 2C. Init INT_count by parsing the i_size data from the picard file for N_0
print STDERR "======== Calculating the insert size for the INT reads aligned to the N_0 INT-Ref =========\n";
my $adjacent_model_refs_insert_size_file = "$tmp_optimal_ref_dir/adjacent_model_refs\_std_insert.metrics";
my @INT_i_sizes;
open( my $int_N0_picard_fh, "<", "$adjacent_model_refs_insert_size_file" )
    or confess "Error: Unable to open picard_file for reading: $adjacent_model_refs_insert_size_file\n";
## Start reading the INT i_size data from the merged_N0_reference.bam picard file
my $header_2 = 1;
while (<$int_N0_picard_fh>) {
    chomp( my $picard_data_line = $_ );
    if ( $picard_data_line =~ /^insert_size/ ) { $header_2 = 0; next; }
    elsif ( $header_2 == 1 ) { next; }
    elsif ( $picard_data_line =~ /^\d+/ ) {
        my ( $i_size, $fr, $rf, $tandem ) = split( /\t/, $_ );
        for ( my $i = 1; $i <= $fr; $i++ ) {
```

```
            push( @INT_i_sizes, $i_size );
        }
    }
}
close $int_N0_picard_fh;

# 3. Titrate the optimal distance between the two sides of the INT
## Open the output we will print the AD & JSD calculations to
open( VAR, ">", "$opts->{output_dir}\/Variance_from_avg.txt" )
    or confess "Error: Unable to open output file: $opts->{output_dir}/Variance_from_avg.tx";
## Print Header
printf VAR ( "%-20s%-20s", "N", "Variance_from_avg" );
## JSD calculation header
if ( $jsd == 1 ) { printf VAR ( "%-20s%-20s%-20s", "JSD", 'ci_min', 'ci_max' ); }
print VAR "\n";

# Local hash variables to store the titration data
my %AD_titration;     ##  AD{$N} = calc_AD_at_N
my %jsd_titration;    ## JSD{$N} = calc_JSD_at_N

# Titrate 0-100 bp between the INT refs, calculate the AD & JSD.
print STDERR "======== Titrating the optimal distance between the consensus sequences =========\n";
for ( my $N = 0; $N <= 100; $N++ ) {
    my @diff_N_list;    ## difference between the LIB_median_i_size and each INT_read_i_size w/ #_N's bp between the refs
    my %INT_count;      ## Same data structure as LIB_count
    foreach my $insert (@INT_i_sizes) {
        $INT_count{ ( $insert + $N ) }++;
        push( @diff_N_list, ( abs( $insert + $N - $LIB_median_insert_size ) ) );
    }
    ## Calculate the Average Difference
    my $avg_diff_N = Math::NumberCruncher::Mean( \@diff_N_list );
    $AD_titration{$N} = $avg_diff_N;
    printf VAR ( "%-20s%-20.3f", $N, $avg_diff_N );

    ## Calculate the Jensen-Shannon Distance
    if ( $jsd == 1 ) {
        ## Load functions into R to calculate the JSD
        my $R = Statistics::R->new( r_bin => '/usr/local/bin/R' );
        $R->run('require(boot)');
        $R->run(
            'calc_JSD <- function(inMatrix, pseudocount=0.0000001, ...) {
                            KLD <- function(x,y) { sum(x *log(x/y)) }
                            JSD <- function(x,y) { sqrt(0.5 * KLD(x, (x+y)/2) + 0.5 * KLD(y, (x+y)/2)) }
                            matrixColSize <- length( colnames( inMatrix ) )
                            matrixRowSize <- length( rownames( inMatrix ) )
                            colnames <- colnames( inMatrix )
```

```
                    resultsMatrix <- matrix( 0, matrixColSize, matrixColSize )

                    inMatrix = apply( inMatrix, 1:2, function(x) ifelse ( x==0, pseudocount, x ) )
                    for ( i in 1:matrixColSize ) {
                        for ( j in 1:matrixColSize ) {
                            resultsMatrix[ i, j ] = JSD( as.vector( inMatrix[ , i ] ), as.vector( inMatrix[ , j ] ) )
                        }
                    }
                    colnames -> colnames( resultsMatrix ) -> rownames( resultsMatrix )
                    as.dist( resultsMatrix ) -> resultsMatrix
                    attr( resultsMatrix, "method" ) <- "dist"
                    return( resultsMatrix )
                }'
);

$R->run(
    'calc_JSD_boot_fxn <- function (x_df, index) {
                tmp_df <- data.frame(x_df[index,])
                return( calc_JSD(tmp_df) )
                }'
);

## Initialize R data.frame with LIB & INT count of i_size in R
$R->run('LIB_count = numeric()');
$R->run('INT_count = numeric()');
foreach my $key ( sort { $a <=> $b } keys %LIB_count ) {
    my $LIB_count_at_N_key = $LIB_count{$key};
    my $INT_count_at_N_key = defined $INT_count{$key} ? $INT_count{$key} : "0.0000001";    ## JSD can't have counts=0
    $R->run("LIB_count = c( LIB_count, $LIB_count_at_N_key )");
    $R->run("INT_count = c( INT_count, $INT_count_at_N_key )");
}
$R->run('counts=data.frame(LIB_count,INT_count)');
## Calculate the proportion of each i_size in LIB & INT
$R->run('ct=prop.table(as.matrix(counts), margin=2)');

## Calculate the Jensen-Shannon Distance & parse output
my $JSD_lines = $R->run('calc_JSD(ct)');
my @JSD_split = split( /\n/, $JSD_lines );
my $calc_JSD  = ( split /\s+/, $JSD_split[1] )[1];
$jsd_titration{$N} = $calc_JSD;
printf VAR ( "%-20.5f", $calc_JSD );

# Calculate the JSD confidence interval for the model & parse output
my $jsd_ci_lower;
my $jsd_ci_upper;
## Bootstrap the INT population while keeping the LIB population_freq intact
```

```
        $R->run("JSD_boot <- boot(ct, calc_JSD_boot_fxn, R=1000, stype = \"i\", parallel=\"multicore\", ncpus=$threads)");
        my $JSdist_ci = $R->run('boot.ci(JSD_boot, type="norm")');
        my $ci_data_line = ( split /\n/, $JSdist_ci )[8];
        if ( defined $ci_data_line ) {
            $ci_data_line =~ /\s+\((.+)\,\s+(.+)\)/;
            $jsd_ci_lower = $1;
            $jsd_ci_upper = $2;
            printf VAR ( "%-20.4f%-20.4f", $jsd_ci_lower, $jsd_ci_upper );
        }
        else {
            $jsd_ci_lower = "NULL";
            $jsd_ci_upper = "NULL";
            printf VAR ( "%-20s%-20s", $jsd_ci_lower, $jsd_ci_upper );
        }

        ## Close R instance
        $R->stop();
    }
    print VAR "\n";
}
close VAR;

# Determine the optimal distance between the two sides of the INT
my $opti_AD_N = &find_key_with_min_hash_value( \%AD_titration );                              ## Optimal AD distance
my $opti_JSD_N = ( $jsd == 1 ) ? &find_key_with_min_hash_value( \%jsd_titration ) : undef;    ## Optimal JSD distance

# Print the optimal distance
open( OPT, ">", "$opts->{output_dir}/Opti_dist.txt" )
    || confess "Error: Unable to open file to record optimal distance between references: $opts->{output_dir}/Opti_dist.txt\n";
print OPT "Opti_Distance: $opti_AD_N";
if ( $jsd == 1 ) { print OPT "\tJSD_Distance: $opti_JSD_N | JSD_value: $jsd_titration{$opti_JSD_N}"; }
print OPT "\n";
close OPT;

# Graph the AD & JSD titration data
my $R = Statistics::R->new( r_bin => '/usr/local/bin/R' );
$R->run("Table=read.table(\"$opts->{output_dir}\/Variance_from_avg.txt\", header=T, row.names=1)");
$R->run('df=data.frame(x=seq(0,length(Table[,2])-1), diff=Table[,1], jsd=Table[,2], lwr=Table[,3], upr=Table[,4])');
if ( $jsd == 1 ) {
    ## Create the JSD plot
    $R->run("pdf(file=\"$opts->{output_dir}\/JSD_plot.pdf\")");
    $R->run('plot( jsd~x, data=df, ylim=range(c(df$lwr,df$upr)), cex=.1)');
    $R->run('with( df, polygon(c(x,rev(x)), c(lwr,rev(upr)), col="grey75", border=FALSE))');
    $R->run('matlines( df[,1], df[,c(-1,-2)], lwd=c(4,2,2), lty=1, col=c("black","red","red"))');
    $R->run("abline( h=$jsd_titration{$opti_JSD_N}, col=\"magenta\")");
    $R->run("abline( v=$opti_JSD_N, col=\"magenta\")");
```

```
        $R->run('dev.off()');
    }
    ## Create the AD plot
    $R->run("pdf(file=\"$opts->{output_dir}\/AD_plot.pdf\")");
    $R->run('plot(diff~x, data=df, ylim=range(df$diff), cex=.3, pch=19, cex.axis=0.6, cex.lab=0.6, font=2)');
    $R->run("abline( h=$AD_titration{$opti_AD_N}, col=\"magenta\")");
    $R->run("abline( v=$opti_AD_N, col=\"magenta\")");
    $R->run('dev.off()');

    # Close R instance
    $R->stop();

    if ( $opts->{titrate_n_string} == 1 ) {
        my @stdev_titration = ( 2, 1, .5, 0, -.5, -1 );
        foreach my $deviation (@stdev_titration) {
            my $step
                = ( $jsd == 1 )
                ? ( $opti_JSD_N + ( $LIB_stdev * $deviation ) )
                : ( $opti_AD_N + ( $LIB_stdev * $deviation ) );
            if ( $step >= 0 ) { push( @n_num_list, $step ); }
        }
    }

    print STDERR "======== Optimal reference calculated ========\n";
    run_cmd("rm -rf $tmp_optimal_ref_dir");
    return ( \@ret_ref1_data_list, \@ret_ref2_data_list, @n_num_list );
}
```
