# Supplementary material for: Modeling the integration of bacterial rRNA fragments into the human cancer genome
Source: BMC Bioinformatics. 2016 Mar 21;17:134. doi: 10.1186/s12859-016-0982-0 (PMC4802584; doi:10.1186/s12859-016-0982-0)
Supplement: Additional file 10: Table S1 — Conversion of the participant names to SRR accession number. (DOC 66 kb) [file 12859_2016_982_MOESM10_ESM.doc]

## Table S1. Conversion of the participant names to SRR accession number

| **Participant A** | SRR203142 | SRR203143 |
| --- | --- | --- |
| **Participant B** | SRR203140 | SRR203141 |
| **Participant C** | SRR203156 | SRR203157 |
| **Participant D** | SRR203160 | SRR203161 |
| **Participant E** | SRR203150 |  |
| **Participant F** | SRR203146 | SRR203147 |
